# C-Reactive Protein Is an Important Biomarker for Prognosis Tumor Recurrence and Treatment Response in Adult Solid Tumors: A Systematic Review

**DOI:** 10.1371/journal.pone.0143080

**Published:** 2015-12-30

**Authors:** Shiva Shrotriya, Declan Walsh, Nabila Bennani-Baiti, Shirley Thomas, Cliona Lorton

**Affiliations:** 1 Department of Solid Tumor Oncology, The Cleveland Clinic Taussig Cancer Institute, Cleveland, Ohio, United States of America; 2 The Harry R. Horvitz Center for Palliative Medicine, The Cleveland Clinic Taussig Cancer Institute, Cleveland, Ohio, United States of America; 3 Our Lady’s Hospice & Care Services, Harold’s Cross, Dublin, Ireland; University Medical Center of Princeton/Rutgers Robert Wood Johnson Medical School, UNITED STATES

## Abstract

**Purpose:**

A systematic literature review was done to determine the relationship between elevated CRP and prognosis in people with solid tumors. C-reactive protein (CRP) is a serum acute phase reactant and a well-established inflammatory marker. We also examined the role of CRP to predict treatment response and tumor recurrence.

**Methods:**

MeSH (Medical Subject Heading) terms were used to search multiple electronic databases (PubMed, EMBASE, Web of Science, SCOPUS, EBM-Cochrane). Two independent reviewers selected research papers. We also included a quality Assessment (QA) score. Reports with QA scores <50% were excluded. PRISMA (Preferred Reporting Items for Systematic Reviews and Meta-Analysis) methodology was utilized for this review (S1 PRISMA Checklist).

**Results:**

271 articles were identified for final review. There were 45% prospective studies and 52% retrospective. 264 had intermediate QA score (≥50% but <80%); Seven were adequate (80% -100%); A high CRP was predictive of prognosis in 90% (245/271) of studies—80% of the 245 studies by multivariate analysis, 20% by univariate analysis. Many (52%) of the articles were about gastrointestinal malignancies (GI) or kidney malignancies. A high CRP was prognostic in 90% (127 of 141) of the reports in those groups of tumors. CRP was also prognostic in most reports in other solid tumors primary sites.

**Conclusions:**

A high CRP was associated with higher mortality in 90% of reports in people with solid tumors primary sites. This was particularly notable in GI malignancies and kidney malignancies. In other solid tumors (lung, pancreas, hepatocellular cancer, and bladder) an elevated CRP also predicted prognosis. In addition there is also evidence to support the use of CRP to help decide treatment response and identify tumor recurrence. Better designed large scale studies should be conducted to examine these issues more comprehensively.

## Introduction

Approximately 1,638,910 new cancer diagnoses and about 577,190 deaths occurred in the US in 2012, mostly from solid tumors [1]. Prognostication in cancer can be either subjective or objective. In the former, dependent on clinician skill and experience, it is often inaccurate and usually overly optimistic [2]. Prognostication is an important clinical skill for oncologists. Despite advances in medical technology and biology, it is still an inexact science [2], even with extensive and expensive investigations [3]. Objective determination of prognosis can be based on a combination of tumor, patient, and environmental factors. The use of biological tumor markers to help prognostication (alone or combined with other parameters) has appeal. An ideal potential tumor marker should have a long half-life, be measured accurately and precisely by a simple and inexpensive blood test. It is also important that it be sensitive to change so that it can be followed over time through serial measurements. A few biologic markers meet these criteria [4]. C-reactive protein (CRP) is one.

### Rationale

CRP is an acute phase reactant, which reflects tissue injury [5]. The half-life is 19 hours in both health and disease. CRP secretion by hepatocytes appears controlled by interleukin 6 (IL-6). Interleukin-1 (IL-1) and tumor necrosis factor (TNF) also stimulate CRP synthesis [6]. CRP is a stable downstream marker of inflammation, unlike the pro-inflammatory cytokines, which have short half-lives (minutes) [7, 8]. In chronic inflammatory diseases, serial CRP levels have been correlated with disease severity, and response to therapy [9]. Many large prospective studies now support the role of CRP in prediction of coronary artery disease [10, 11], though controversies exist [12].

Chronic inflammation has been linked to cancer at tumor initiation, but may also be associated with invasive potential and disease progression [13, 14]. A relationship has been proposed between systemic inflammation and various cancer symptoms [15]. A strong positive correlation between high CRP and high IL-6 levels was shown in advanced pancreatic cancer [16]. Elevated CRP levels have been linked to shorter survival in several common cancers [17].

### Objectives

In this paper, we describe the results of a systematic review of the relationship between elevated serum CRP and life expectancy in people with solid tumors. We also examined its role in the prediction of treatment response and risk of tumor recurrence.

## Methods

### Eligibility criteria

Only articles in English were included. Original reports of any studies of solid malignancies in adults were scrutinized. All study designs were included. The following articles were excluded: all non-English literature, basic research, animal research, all pediatric and hematological malignancies, and studies where prognostic parameters were not assessed, or serum CRP levels not measured. Editorial letters and comments were also excluded. Review papers were consulted, but for discussion purposes only.

### Information sources

Electronic databases included: PubMed (1966 to December 2012); EMBASE (1988 to 2012); Web of Science (1980 to 2012); SCOPUS (1965 to 2012); and the EBM-Cochrane Central Register of Controlled trials and EBM-Cochrane Database of Systematic Reviews (Up to 2012). The search was repeated at the end of data analysis.

### Search

PubMed search of CRP or related MeSH terms (c—reactive protein/c-reactive) with (AND) neoplasm/neoplasms/cancer in all fields with (AND) prognosis/mortality/survival OR survival rate/treatment outcome/treatment/outcome was done including other databases. Search words/terms were as follows:

### Study selection

**Fig 1:**
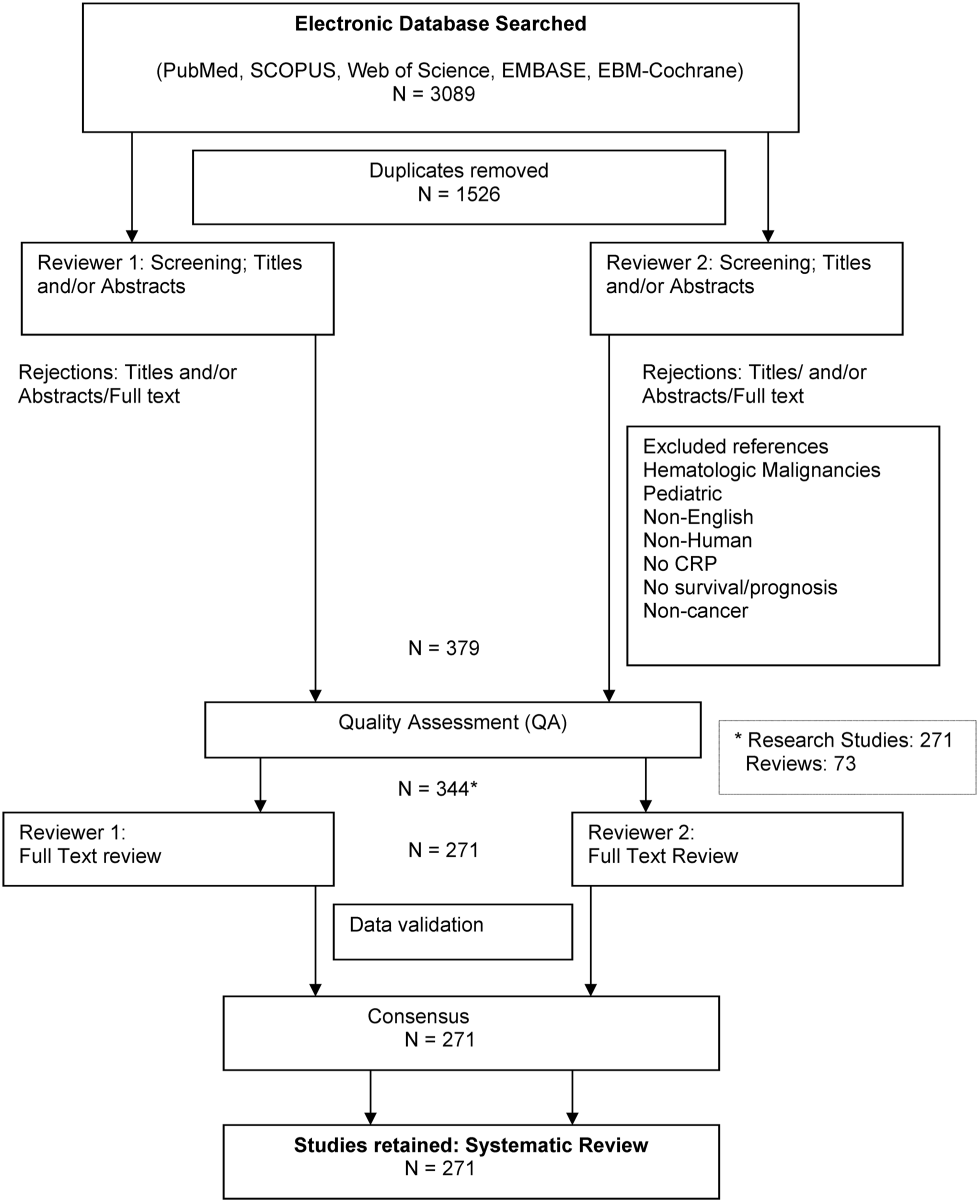
Studies Selection.

A qualified medical librarian (see Acknowledgements) reviewed the search strategy. The first literature screen (Fig 1) was based on article title. If that was irrelevant, the abstract was also reviewed (by NBB, SS and DW) before an exclusion decision. Abstracts (and when necessary the full text) of the remaining articles were then assessed. The reviewers (NBB, SS, ST and DW) met periodically to discuss reasons of exclusion or inclusion of selected papers. Retained articles were then subjected to quality assessment (S1 Appendix).

### Quality assessment

A quality assessment (QA) system was developed. This was based on existing guidelines [18, 19] for observational cohort prognostic studies. The tool combined five criteria:

1. Study design
2. Patient selection
3. Prognostic variables
4. Follow-up
5. Data analysis

A score of 0–2 was assigned to each—if a study met the conditions in full (score of 2), partially (score of 1), or not at all (score of 0) (S1 Appendix). The total was expressed as a percentage of the maximum possible score. A score of 80–100% was ranked as an adequate study; ≥50 but <80% as intermediate; articles that scored <50% were considered inadequate and excluded from the review (S2 Appendix).

### Data collection

Data were extracted using a custom designed extraction sheet (till 2010) and later utilizing the Research Electronic Data Capture (REDCap) [20] forms with same sheet. REDCap is a secure web application to create and manage data. The following information was collected: author name, publication date, study title, study design, quality assessment score and grade, number of patients and controls (when available), cancer type, extent of disease, main outcome, main results related to prognosis or treatment outcomes, CRP cut-off points, assay method, mean CRP value, survival definition, median survival duration, CRP sensitivity and specificity as a predictor of prognosis, treatment outcomes or recurrence, other parameters assessed for prognosis, strongest predictors of prognosis, statistical analysis used, and possible co-morbid contributors to increased CRP levels other than cancer (like infection, chemo- or radiotherapy, surgery). The descriptor term “cutoff” refers to the value an investigator/author used to determine an “elevated” CRP even if that level was within the biochemical reference range.

### Summary measures

There was substantial variation in study design and cancer primary sites. A meta-analysis was therefore inappropriate. For each study article we estimated the minimum sample size necessary to detect a difference at P ≤ 0.05. We used the general rule of n = 10 per variable. The estimated minimum sample size was compared to the actual size of the study. Studies with insufficient sample sizes were considered underpowered. Predictors by multivariate analysis were stratified by relative risk (RR) and statistical significance (p-value):

1. When RR<2 or >0.5
2. When RR>2 or <0.5
3. When RR>5 or <0.2
4. When RR>10 or <0.1
5. When p<0.05
6. When p<0.01
7. When p<0.001

We followed the PRISMA statement (S1 PRISMA Checklist) to design and report our systematic review [21].

## Results

### Study selection

The search identified three thousand and eighty nine (3089) citations: fourteen hundred sixty-six (1466) in PubMed, eight hundred two (802) in Web of Science, three hundred twenty (320) in SCOPUS, three hundred eleven (311) in EMBASE and one hundred ninety (190) in the Cochrane database. After removal of duplicates, fifteen hundred twenty six (1526) remained. Irrelevant studies were then removed. These included those where survival or prognosis was not an outcome, studies where CRP was not studied as a prognostic marker, animal/cell-line based studies, letters and editorials, and those that did not fit our inclusion criteria. Seven hundred thirty one (731) papers were left. Next, three hundred (300) studies in hematologic malignancies, ten (10) non-English articles and forty two (42) pediatric reports were removed. Subsequent to the quality assessment (QA) of the three hundred seventy nine (379) studies retained, thirty five (ten prospective, twenty five retrospective) were inadequate by QA score and excluded. Then two hundred seventy one (271) research studies and seventy-three (73) review papers remained (Fig 1). Survival and outcome measures differed between studies. As a result, no direct study comparisons were possible.

Two hundred seventy one original articles constituted the final analysis (Fig 1). Only seven of these scored ≥ 80% in the QA (all were prospective and longitudinal, and with a control group in three). Two hundred sixty four (264) had an intermediate QA score. One hundred twenty nine (129) of the 271 did not describe their patient selection procedures. Examples included whether patients were screened for infections, the timing of CRP measurement in relation to factors that could raise CRP level (like chemo- or radiation therapy), and invasive procedures. The sensitivity and specificity of the predictive prognostic value of CRP were reported in only four studies [22–25], two in melanoma, one each in cancer of the esophagus and lung. A power analysis was described in two [26, 27]; CRP was an independent prognostic marker of survival in one but not the other. The reference level of CRP for evaluation of responses varied both for RCC and GI studies.

### Study characteristics by study design

Forty five percent (n = 122) of the studies were prospective and 52% (N = 142) of the 271 studies were retrospective; the remaining 3% (N = 7) combined retrospective and prospective design. In the prospective studies, median sample size was 121 (range 15–9605) versus 146 (range 32–9608) in the retrospective. High CRP predicted prognosis in 82% (100/122) of the prospective studies. In 13% (16/122) of prospective studies, this was by univariate analysis only. In 18% (22/122), CRP was not prognostic of survival (Table 1). Only 16% (20 of 122) of the prospective studies had a control group (CG) (Table 1). Overall CRP predicted prognosis in 90% (245/271) of studies; 80% by multivariate analysis (MVA) and 20% by univariate analysis (UVA) (Table 2).

**Table 1:** Characteristics by Study Design.

Study Type	Number of Studies	Sample Size		Study Outcomes^#^ (%)		
	(%)*	Median	Range	1	2	3
**Prospective**	122 (45)	121	(15–9605)	100 (82)	22 (18)	16 (13)
No control group	102 (84)	117	(15–9605)			
Control group	20 (16)	156	(54–687)			
**Retrospective**	142 (52)	146	(32–9608)	128 (90)	14 (10)	29 (20)
**Combined** ^+^	7 (3)	98	(58–325)	7 (100)	0	0

**1**: Number of Studies Where CRP was a Prognostic Predictor.

**2**: Number of Studies Where CRP was Not a Prognostic Predictor.

**3**: Number of Studies Where CRP was a Prognostic Predictor by Univariate Analysis Only.

* Percent (rounded to the closest whole number) compared to total number of studies.

^**#**^ Percent compared to study type.

^**+**^ Both prospective and retrospective data.

**Table 2:** Study Characteristics by Tumor Type.

Cancer Type	Number of Studies	Study Outcomes		
		1 (%)	2 (%)	3 (%)
Digestive Tumors*	90	81 (90)	9 (10)	16 (18)
Renal cell carcinoma	51	46 (90)	5 (10)	12 (24)
Pancreas	24	23 (96)	1 (4)	7 (29)
Lung	24	22 (92)	2 (8)	2 (8)
Hepatocellular carcinoma (HCC)	10	10 (100)	0 (0)	1 (10)
Melanoma	5	5 (100)	0 (0)	0 (0)
Breast	7	4 (57)	3 (43)	0 (0)
Prostate	9	7 (78)	2 (22)	0 (0)
Bladder	12	12 (100)	0 (0)	2 (17)
Heterogeneous	15	14 (93)	1 (7)	2 (13)
Others	24	21 (88)	3 (13)	6 (25)

**1**: Number of Studies Where CRP was a Prognostic Predictor.

**2**: Number of Studies Where CRP was Not a Prognostic Predictor.

**3**: Number of Studies Where CRP was a Prognostic Predictor on Univariate Analysis Only.

* Digestive tumors include esophageal, gastroesophageal and intestinal tumors.

### Study characteristics by tumor type

#### 1. Renal cell carcinoma

**Fig 2:**
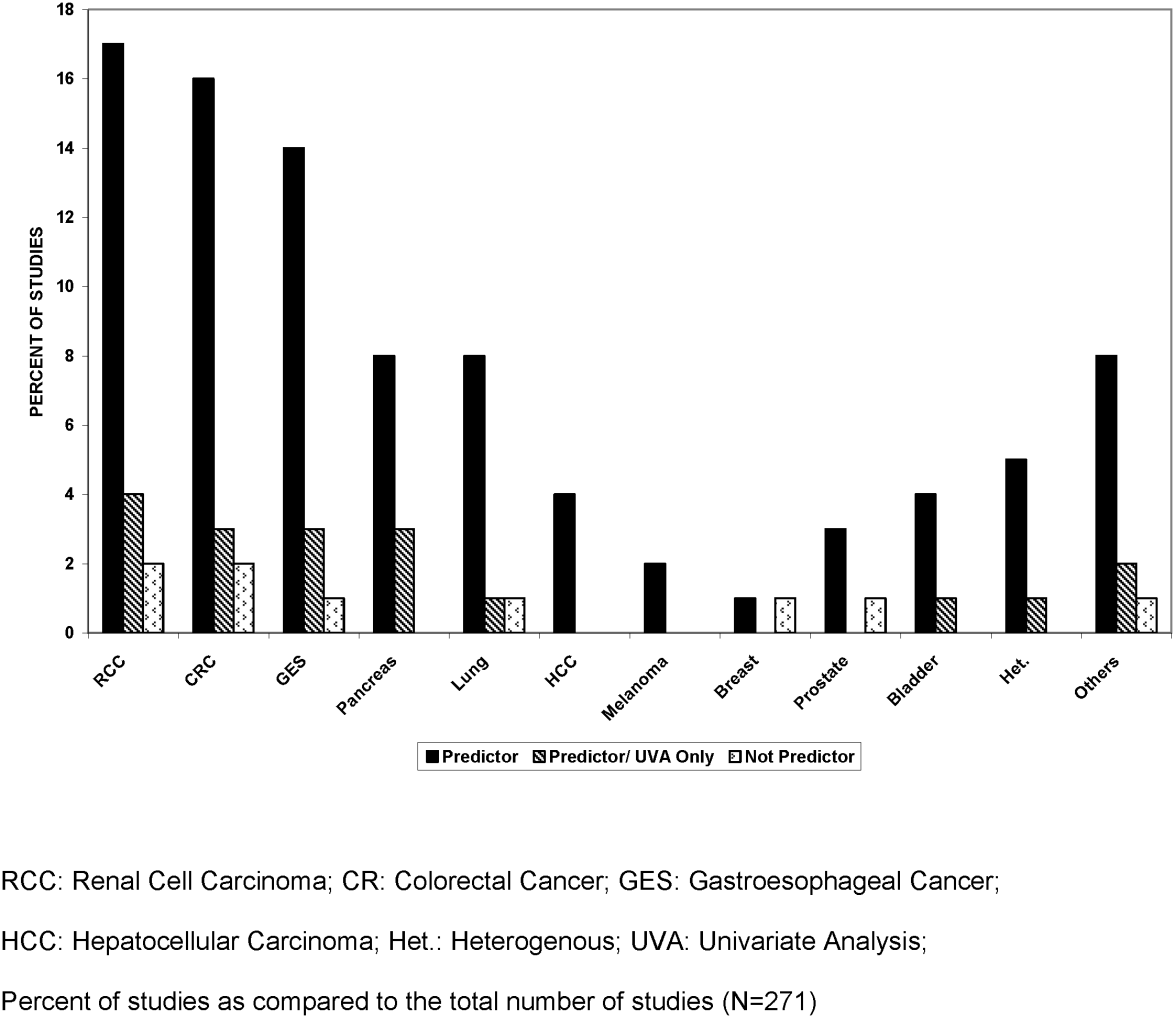
CRP Prediction of Prognosis by Tumor Primary Site.

Fifty one (19%) studies looked at renal cell carcinoma. Of these, CRP was prognostic in 90% (46 of 51). In 12 of 51 (24%), CRP predicted prognosis on univariate analysis only [28–37]. CRP was not predictive of prognosis in five studies [38] (Table 2, Fig 2).

1.1 Prognosis: Forty-six of the 51 studies in renal cell carcinoma (90%) had prognosis as a primary outcome. In thirty three of the 46, CRP was a strong predictor of survival by multivariate analysis (Table 3). In the other 12 of the 46, CRP predicted prognosis by univariate analysis only [28–37]. One of these was underpowered [39]; none of the other eleven studied the prognostic value of CRP as a primary outcome measure (Table 3).

**Table 3:** CRP as a Predictor of Prognosis, Treatment Outcome or Tumor Recurrence in Renal Cell Carcinoma.

Publication Year (Reference)	Main Outcome	CRP cut-offs* (mg/L)^#^	Study Design	Quality Score (%)	Sample Size	Disease Extent	Strongest Predictors by MVA^ϕ^	
**PROGNOSIS**								
**1998** [144]	5 year Survival Post Curative Resection	Negative vs. positive	Retrospective	55	111	All stages	CRP	b, x
							T stage	a, x
**1999** [145]	Pre-treatment Serum Markers and Clinical Parameters	≥8	Prospective	65	99	Metastatic	SICAM-1	—, y
							CRP	—, x
							ESR	—, x
**2006** [146]	APP in Potentially Curative Resection	>10	Prospective + Retrospective	60	43 Prospective 57 Retrospective	All stages	CRP	c, y
							Grade	b, y
							Sex	b, x
**2006** [147]	Estimation by Fractional Polynomials	Not reported	Retrospective	65	425	Metastatic	Age	x
							LN, liver, bone metastasis	—, y,x,y
							CRP	—, x
							Neutrophils	—, y
**2007** [148]	Preoperative Serum CRP	>5	Prospective	65	101	Localized	DSS: pT staging	b, y
							CRP	b, x
							RFS: CRP	b, z
							pT Staging	b, y
**2007** [149]	GPS and Cancer-specific Survival	>10	Prospective	70	119	Metastatic	Biochemical: Calcium	b, y
							CRP	b, y
							Albumin	b, y
							WCC	a, y
							Scoring systems: GPS	b, z
							MSKCC	a, y
**2007** [150]	Stages Treated with Nephrectomy–Survival UISS v. Model with CRP	Continuous Categorical: ≤4.0, 4.1–23.0, >23.0	Prospective	65	313	All Stages	CRP (Cat.)	—, y
							Metastasis	b, z
							ECOG PS	b, y
**2007** [151]	Survival, Treatment Response: IL-2 Based Therapy	8mg/L	Retrospective With Control	60	55 + 144 Controls	Metastatic	CRP	b, y
							IL-12	a, x
**2008** [50]	Survival: Primary Operable Tumor Recurrence	>10mg/L	Prospective	75	83	All Stages	CRP	d, y
							T-stage	d, x
							Necrosis	d, y
**2008** [33]	Prognosis: RCC Extending IVC	6mg/L	Retrospective	55	46	All Stages	CRP	b, —
							LN Metastasis	b, y
**2008** [51]	CRP, Tumoral IL-6, COX-2 Expression & Survival	10mg/L	Retrospective	60	60	Resectable	CRP	b, x
							TNM	c, x
**2008** [152]	Systemic Symptoms on Survival	3mg/L	Retrospective	55	252	All Stages	CRP	b, y
							Systemic Symptoms	c, z
**2008** [123]	Survival in Cytoreductive Nephrectomy	5mg/L	Prospective	65	40	Metastatic	CRP kinetics	b, z
							Poor ECOG	—, y
							Number of Mastectomy	—, y
							Bone Metastasis	—, x
**2009** [122]	CRP Kinetics & Survival	Normalized & Non-Normalized	Retrospective	60	108	Metastatic	Normal CRP	a, x
							Non-Normal CRP	b, z
							ECOG PS	a, z
							LDH	a, z
**2009** [153]	Survival Prediction Model with CRP	5mg/L	Prospective with Control	75	249 Control-290	Locally Advanced	CRP	a, x
							Distant Metastasis	b, z
**2009** [32]	Worst Grade Component Survival, Recurrence	≥10 mg/L	Retrospective	50	314	All Stages	CRP	a,—
							Distant Metastasis	c, z
**2010** [53]	Preoperative CRP Survival, Metastasis	Continuous	Prospective	85	130	All Stages	Pre-operative CRP	a, z
							Pre-operative Platelets	a, z
**2010** [49]	Preoperative and Postoperative CRP to Predict Outcome	Continuous	Prospective	70	110	Localized	Post-operative CRP	a, z
							T-stage	d, —
**2010** [48]	Pre-operative Prognostic Significance of CRP	15mg/L	Retrospective	60	286	All Stages	Log (CRP)	a, y
							M-Stage	b, z
							Necrosis	a, y
							MVI (invasion)	b, y
							RBC	b, y
							WBC	b, z
**2011** [37]	Lifestyle Factors on CRP and Overall Survival	2mg/L	Prospective	70	257	Localized	Pre-operative CRP	a, —
**2011** [54]	CRP and Thrombocytosis on Survival	8mg/L	Retrospective	55	177	Resectable	CRP	b, x
							Tumor size	b, y
**2011** [154]	CRP on Survival, Predictive Survival Model	3mg/L	Retrospective	50	94	Metastasis (Bone)	CRP	b, x
							Sarcoma Differentiate	b, z
							Bone Involvement	b, y
							Extraosseus Metastasis	b, x
							ALP	c, x
**2011** [55]	CRP, CRP Kinetics: Survival and Recurrence	10mg/L	Retrospective	55	263	Resectable	Non-normal CRP	—, z
							Anemia	—,—
							Thrombocytosis	—,-
**2011** [155]	Prognosis of Metastatic RCC; Validity of MSKCC	3mg/L	Retrospective	50	473	Metastasis	CRP	b, z
							Diagnosis→Metastasis (Time)	b, z
							Hemoglobin	a, y
							Calcium	a, y
							LDH	a, x
							Liver metastasis	b, z
							Bone metastasis	a, y
							Node metastasis	a, y
**2011** [56]	Factors Associated WithSurvival, Recurrence	4 mg/L	Retrospective	50	32	Metastasis	CRP	b, x
**2011** [156]	Post-operative CRP, pre-operative albumin and survival	2 mg/L	Retrospective	50	40	Resectable	Postoperative CRP	a, x
							Preoperative Albumin	c, x
**2012** [157]	mGPS and Prognosis	10 mg/L (mGPS)	Prospective	70	169	All Stages	mGPS	b, z
							Necrosis	a, x
**2012** [158]	Systemic inflammation, Tumor inflammatory cells, Tumor Necrosis & Survival	10mg/ L (mGPS)	Prospective	60	79	Resectable	mGPS	c, z
**2012** [159]	Survival & Treatment Response with Sunitinib	3mg/L	Retrospective	50	41	All Stages	Elevated CRP	—/—
							Normal CRP	d, x
**2012** [160]	Molecular-targeted agents, Survival & Treatment Response	8mg/L	Retrospective	55	52	Metastasis	CRP	a, y
							Neutrophilia	a, x
**2012** [161]	Hyponatremia on Survival with Molecular Targeted Therapy	10mg/L	Retrospective	50	87	Metastasis	Severe Hyponatremia	b, x
							Mild Hyponatremia	c, z
							CRP	a, y
							Neutrophilia	b, x
**2012** [162]	WBC, CRP and Survival, Optimal Threshold of CRP	25mg/L	Retrospective	55	327	Resectable	CRP	b, x
							T stage	b, x
							N stage	b, y
							M stage	c, z
							Nuclear grade	b, z
							Karnofsky	b, y
**2012** [163]	Prognostic significance of Osteopontin A, Carbonic Anhydrase IX, CRP; alone and combined	Continuous	Retrospective	55	216	All Stages	CRP	a, z
							CA-9	b, y
							N stage	b, z
							M stage	b, z
**2012** [164]	Pre-operative CRP	Continuous Categorical: <4mg/L, 4-10mg/L, >10mg/L	Retrospective	55	1161	All Stages	Metastasis	b, z
							G4 Differentiation	b, y
							CRP (Continuous)	a, z
							CRP (Categorical)	a, z
							CRP (Categorical)	b, z
**TREATMENT RESPONSE AND TUMOR RECURRENCE**								
**1992** [40]	CRP and IL-2 Response	>10	Prospective	60	15	Metastatic	CRP	—
**1992** [41]	Serum IL-6, pre-IL-2	>50	Prospective	50	138+ 70 controls	Metastatic	CRP	—
							IL-6	—
**1999** [42]	Cytoreductive Surgery Subgroups	≥1ng/ml	Retrospective	50	58	Metastatic	CRP	—
**2003** [43]	Prognostic System Post-IL-2 + INF-α	≥11	Retrospective	55	425	Metastatic	WBC	a, z
							CRP	a, y
							LDH	a, x
							Number of Metastasis	a, x
							Time to Metastasis	a, y
**2004** [44]	APP, Performance Status and Survival post-IFN-α	≤10 vs. >10	Prospective+ Retrospective	55	26 Retrospective 32 Prospective	Advanced	CRP	b, x
**2005** [45]	Pre-treatment (IL-2) Biohumoral and Clinical Factors	≥8	Retrospective	60	110	Metastatic	CRP	b, x
							DFI	b, x
**2005** [46]	Prognostic Factors Post-Allogeneic Stem Cell Transplant	Normal or not	Prospective	65	70	Advanced	CRP	b, z
							LDH	b, x
							KPS	a, x
**2006** [47]	Response and Survival Post IFN-α then IL-2.	≥8	Retrospective	55	99	Metastatic	Nuclear grade	b, y
							Mastectomy	b, x
							LDH	b, x
							CRP	b, x
**2006** [52]	CRP, Thrombocytosis and Recurrence	>10	Retrospective	55	178	All stages	Metastasis	d, z
							CRP	c, z
							Tumor grade	b, x
							Tumor size	a, y
**2008** [50]	Primary Operable Tumor Recurrence	>10mg/L	Prospective	75	83	All Stages	CRP	b, x
							UISS	b, z
							SSIGN	b, x
**2008** [51]	CRP, Tumoral IL-6, COX-2 Expression & Recurrence Free Survival	10mg/L	Retrospective	60	60	Resectable	CRP	b, x
							TNM	b, x
**2009** [32]	Worst Grade Component And Recurrence	≥10 mg/L	Retrospective	50	314	All Stages	CRP	c, x
**2010** [53]	Preoperative CRP and Metastasis	Continuous	Prospective	85	130	All Stages	Pre-operative CRP	—, z
							SSIGN	d,—
**2010** [48]	Pre-operative CRP and Disease Free Survival	15mg/L	Retrospective	60	286	All Stages	Log (CRP)	b, z
							Stage	a, y
							MVI (invasion)	b, z
**2010** [49]	Postoperative CRP to Predict Recurrence	Continuous	Prospective	70	110	Localized	Post-operative CRP	a, z
							T-stage	d, z
**2011** [54]	CRP and Recurrence	8mg/L	Retrospective	55	177	Resectable	CRP	b, x
							Tumor size	b, y
**2011** [55]	Post-nephrectomy CRP, CRP Kinetics & Recurrence	10mg/L	Retrospective	55	263	Resectable	High Pre-operative CRP	-/z
							Non-normal CRP	—, z
							MVI	—/—
							Tumor Necrosis	—/-
**2011** [56]	Risk Factors for Metastasis	4mg/L	Retrospective	50	32	Metastasis	CRP	b, y
							Symptoms	c, z
							Size	b, x
							Histologic Grade	a, y
							Sarcoma Component	d, z
							MVI	a, x
**2011** [156]	Post-operative CRP, Pre-operative Albumin and Recurrence	2mg/L	Retrospective	50	40	Resectable	Post-operative CRP	d, x

* All CRP levels reported in results correspond to serum levels unless otherwise specified.

^#^ Since CRP values are reported in different units, for uniformity purposes we converted all values to mg/L unless otherwise specified.

^ϕ^ Strongest predictors by MVA were stratified by relative risk (RR) and statistical significances (p) as follows:

^a^ RR<2 or >0.5

^b^ RR>2 or <0.5

^c^ RR>5 or <0.2

^d^ RR>10 or <0.1

^x^ p<0.05

^y^ p<0.01

^z^ p<0.001

—values not reported or no MVA

**Abbreviations**: **ALP**: Alkaline Phosphatase; **APP**: Acute Phase Protein(s); **DFI**: Disease Free Interval; **DSS**: Disease Specific Survival; Score; LDH: Lactate Dehydrogenase; **LN**: lymph Node(s); **MP**: Medroxyprogesterone; **MSKCC**: Memorial Sloan-Kettering Cancer Center; **MVI:** Micro vascular Invasion; **MVA**: Multivariate Analysis; **RFS**: Recurrence Free Survival; **RR**: Relative Risk; **SSIGN**: Stage Size Grade Necrosis; **UISS**: University of California Los Angeles Integrated Staging System; **WCC**: White Cell Count.

1.2. Treatment response: Thirteen of the 51 studies in renal cell carcinoma had treatment response and prognosis as a primary outcome [38, 40–51]. In 12 of the thirteen, CRP independently predicted both treatment response and prognosis. Six studies [40, 44, 48–51] investigated CRP and treatment response as a primary outcome. High CRP predicted treatment response in all except one [38]. This study was also underpowered, and the primary objective was not treatment response (Table 3). Treatment responses (ill-defined) were evaluated after resection of localized tumors and after cytokine based therapies (IL-2 infusions, IF-α) in metastatic RCC. Low CRP level was associated with better treatment responses overall in 11 of thirteen studies.

1.3. Tumor recurrence: Six of the 51 renal cell carcinoma studies [32, 52–56] investigated recurrence and survival as primary outcomes. In three studies, elevated CRP independently predicted both tumor recurrence and prognosis [54, 56] (Table 3). One of these [55] examined CRP kinetics (change in CRP over time) and identified non-normalization of postoperative CRP as a predictor of recurrence.

#### 2. Gastrointestinal malignancies

Of 90 studies, 48 were in colorectal and 42 in esophageal, gastric or gastroesophageal cancers. In colorectal cancer, high CRP strongly predicted survival in 36 (75%) studies. High CRP was an independent prognostic indicator in most reports, 31 of 36 (65%). Only two [57, 58] were negative. In another ten studies [26, 59–66] CRP predicted prognosis by univariate analysis only; one of these was underpowered (Table 2, Fig 2). Elevated CRP independently predicted prognosis in thirty five of the 42 (71%) studies in gastroesophageal cancer.

2.1. Prognosis: Most studies (81 of 90) in gastrointestinal malignancies had survival as a study outcome. Eighty percent (65 of 81) investigated CRP and prognosis as the primary outcome. High CRP was an independent predictor of survival in 56% (45 of 81) and a strong predictor in 25% (20 of 81). It was a predictor on univariate analysis only in 20% (16 0f 81). In two studies [26, 67], CRP predicted prognosis (but not independently of disease stage). Once this was considered, in those two, CRP was not a statistically significant prognostic predictor (Table 4) [165–225].

**Table 4:** CRP as a Predictor of Prognosis, Treatment Outcome or Tumor Recurrence in Digestive Tumors.

Publication Year (Reference)	Main Outcome	CRP cut-offs* (mg/L)^#^	Study Design	Quality Score (%)	Sample Size	Disease Extent	Strongest Predictors by MVA^ϕ^	
**PROGNOSIS**								
**Colorectal**								
**1998** [165]	Preoperative CRP and Clinicopathologic Factors	>8	Prospective	65	120	All stages	CRP	—
**2000** [166]	PAI-1 and CRP Post-resection	>9.8	Prospective	70	594	All stages	CRP	a, z
**2003** [167]	Pre-/postoperative CRP in Curative Resection	>10	Prospective	65	174	Dukes’ A, B, C	CSS CRP	c, y
							Dukes	c, x
							Age	a, x
**2003** [168]	Deprivation, CRP in Curative Resection	>10	Prospective	65	174	Dukes’ B, C	CSS: Age	b, z
							Dukes’	b, x
							CRP	b, x
**2004** [169]	Perioperative APP; IL-1,6 network	≥10	Prospective	60	75	All stages	CRP	—
**2004** [170]	CRP in Potentially Curative Resection	>10	Prospective	65	147	Duke's B, C	Dukes	c, z
							CRP	b, z
							Age	a, y
**2004** [171]	PH vs. Laparotomy Effects on Markers in Liver Metastasis	>2	Prospective	70	24 PH + 9 laparotomy	Liver metastasis	DFS: CRP	—, y
							HGF	—, x
**2005** [172]	IL-6, TNFα, CRP in Local Resection	≥7	Prospective	70	74 + 25 controls	All stages	Unclear: CRP	—
							IL-6	—
**2005** [173]	T-lymphocyte Infiltration + Preoperative CRP	>10	Prospective	60	147	Dukes’ B, C	CSS: CRP	b, z
							Stage	b, z
							Age	b, y
**2006** [174]	Nutritional and Inflammatory Status in Palliative Treatment	>10	Prospective	60	51	Advanced	PS	b, x
							GPS	b, x
							Treatment type	a, y
**2006** [175]	CRP in patients receiving adjuvant 5-FU Post- curative Resection	>10	Prospective	60	222	Duke’s A, B, C	No adjuvant chemotherapy CRP	b, x
							Age	a, x
							Adjuvant chemotherapy CRP	c, x
**2007** [131]	GPS: Post Resection	>10	Prospective	75	316	All stages	mGPS	a, y
							Age	a, x
**2007** [176]	Ki-67 Expression, CRP and Survival	10mg/L	Retrospective	60	147	Curative	CRP	b, z
							Dukes	b, y
							Age	a, y
**2007** [177]	mGPS and Prognosis	10mg/L	Prospective	70	233	All Stages	mGPS	b, z
							Platelet	b, x
							Monocyte	b, y
							Neutrophil	b, y
							WBC	a, z
							TNM	a, x
							Age	a, y
**2007** [178]	Pre-operative Score for Prognosis With Liver Metastasis	10mg/L	Prospective with Control	75	560	Resectable	IRT	a, z
							Metastasis Number	b, x
**2007** [179]	GPS and Post operative Mortality Prediction	10mg/L	Retrospective	65	315	All Stages	GPS	a, x
**2008** [180]	Preoperative and Perioperative CRP Levels and Prognosis	5mg/L	Prospective	80	212	All Stages	CRP	c, x
							Differerentiation	b, x
							Stages	c, x
**2008** [181]	Preoperative CRP in CEA Independent Stage I or II CRC	5mg/L	Retrospective	60	300	All Stages	CRP	a, x
**2008** [182]	Preoperative CRP and Prognosis	5mg/L	Retrospective	65	116	All Stages	CRP	d, z
							Stage	b, y
							Poor Differentiation	b, x
**2008** [183]	Pre-treatment Levels of IL-6, CRP	9.7mg/L	Retrospective with control	65	76, C: 35	All Stages	Tumor Residue	—/ y
							CRP	—/ y
							CA 19–9	—/ x
**2008** [184]	Systemic Inflammatory Response (SIR); GPS; Gene Polymorphism	10mg/L (GPS)	Prospective	55	56	Advanced	GPS: 1	d, x
							Albumin	c, y
							Primary Site	c, x
**2009** [185]	Emergency (ER) Presentation, Preoperative mGPS and Survival	10mg/L	Prospective	70	188	Curative	mGPS	b, x
							Presentation, ER/Elective	b, x
**2009** [186]	Systemic Inflammatory Response (SIR) with Liver Metastasis	10mg/L	Retrospective	65	93	Metastasis	CRP	b, x
							Number of Tumors	b, x
							Hepatectomy	b, x
							Lung metastasis	b, x
**2009** [187]	Local (Klintrup and Jass score) v. Systemic Inflammatory Response (mGPS) and Prognosis	10mg/L (mGPS)	Retrospective	60	287	Curative	mGPS	b, z
							Dukes	b, x
							Age	a, x
							Klintrup	b, x
**2009** [188]	mGPS and Survival	10mg/L (mGPS)	Retrospective	60	112	Unresectable	mGPS	c, y
**2010** [189]	Survival Predictors in Stage IV metastasis	<50, 50–150, >150	Retrospective	55	541	Advanced	CRP	a, x
							Chemotherapy	a, z
							PS	b, z
							Hb	a, z
							Weight Loss	b, z
							Anorexia	b, z
							Fatigue	b, z
							Blood Transfusion	b, z
**2010** [190]	Pre-resection GPS and Survival	10mg/L (GPS)	Prospective	65	63	Metastasis	GPS	b, x
							Liver metastasis	b, x
**2010** [191]	Obesity, Insulin Resistance, Inflammation, Angiogenesis and Survival	4.1	Prospective	60	344	All Stages	CRP	a
							VEGF-A	a, x
							Ang-2	a, x
**2010** [192]	Systemic inflammatory Response Before Curative Resection and Survival	10mg/L (mGPS)	Retrospective	55	320	All Stages	mGPS	a, z
							Age	a, z
							Smoking	a, x
							Dukes	a, z
							POSSUM	a, x
**2011** [193]	mGPS and Prognosis, Effect of Adjuvant Chemotherapy	5mg/L (mGPS)	Retrospective	55	219	Specific Stages, Stage II and III	mGPS	c, y
							Pathology	c, y
**2011** [194]	Hsp70, Acute Phase Proteins (CRP, C1 Inhibitor, C3, C9) and Prognosis	4.7mg/L	Retrospective	65	175	All Stages	CRP	b, x
							sHsp70	a, x
**2011** [195]	Pre-operative Comorbidity, Systemic Inflammation and Survival	10mg/L (mGPS)	Retrospective	55	302	All Stages	mGPS	a, z
							Age	a, z
							TNM	a, z
							Peterson	a, y
							ACE-27	a, y
**2011** [63]	CRP & Prognosis: Peritoneal Carcinomatosis + CRC	35mg/L, Other Cutoffs	Retrospective	50	50	Advanced	CRP	—/z
**2012** [196]	Preoperative Thrombocytosis and Survival After Surgery	Continuous	Retrospective	55	453	All Stages	CRP	a, x
							CEA	a, x
							Tumor Number	b, x
							Platelet	a, x
**2012** [197]	GPS in Synchronous and Metachronous Liver Metastasis	10mg/L (GPS)	Retrospective	50	40	All Stages	GPS 2	c, y
							CA19-9	d, z
							CEA	c, y
**2012** [198]	GPS and survival: Undergoing Curative Surgery	10mg/L (GPS)	Retrospective	55	366	Specific Stages, TNM Stage II & III	GPS	b, z
							LN Mets	a, z
							Lymphatic Invasion	b, x
							Invasion depth	b, y
**Esophagus**								
**2003** [199]	Clinical outcomes & Predictors Before Therapy	≥ 5	Retrospective	60	356	All stages	TNM	a, z
							Weight Change	a, x
							CRP	a, x
**2003** [200]	Clinicopathological & the Prognostic Value of Pre-operative CRP	10mg/L	Retrospective	60	150	All Stages	CRP (low vs. high)	a, x
							LN status	b, y
**2005** [201]	Pretreatment CRP in Chemo/radiation	≥6	Prospective	65	67 + 20 controls	All stages	CRP	—, y
**2006** [202]	Preoperative CRP in Adeno- and Squamous Cell Carcinoma Post-Resection	≥50	Prospective	60	291	All stages	pT stage	—, z
							CRP	a, x
							R classification	—, x
							Transthoracic approach	a, x
**2006** [203]	Clinico-pathological Status & Preop. CRP	>5 and >10	Prospective	70	120	All stages	CRP>10	b, z
							LN metastases	b, z
**2008** [204]	GPS and Survival Prior nCRT	10mg/L (GPS)	Retrospective	70	48	Specific Stages, Stage II and III	GPS	a, y
**2009** [205]	Biomarkers and Survival	<5mg/L, ≥ 5mg/L	Prospective	65	123	All Stages	CRP	d, z
							Treatment	b, y
							Albumin	b, z
**2010** [27]	nCRT Followed by Surgery	8mg/L, 10mg/L	Prospective with Control	70	90, C: 105	Resectable	CRP	c, z
							UICC	b, y
							Radicality	c, z
**2010** [206]	GPS and Survival in Oesophageal Carcinoma (SCC)	10mg/L	Prospective	75	65	Locally Advanced	GPS	a, y
							LN Number	a, x
							Curability	a, x
**2011** [207]	Locally Advanced Disease Undergoing Induction CRT	3mg/L	Retrospective	55	34	Advanced	High CRP (After Chemotherapy)	-/ x
**2011** [208]	GPS in Homogenous Esophageal Cancer	10mg/L (GPS)	Retrospective	65	495	esectable	SCC: GPS1	a, z
							GPS2	b, y
							Adeno: GPS1	a, y
							GPS2	b, z
**2011** [209]	Inflammatory Markers Surgical Resection & Prognosis	10mg/L mGPS	Retrospective	55	112	Resectable	+ LN Ratio	b, z
							mGPS	b, z
**2012** [210]	Local/Systemic Inflammatory Response & Survival	10mg/L mGPS	Prospective	60	121	All Stages	mGPS	d, z
							+ LN ratio	b, z
							CD68 (K-M Score)	a, x
**2012** [129]	CRP and Albumin & Risk stratification	5 mg/L (Fuzzy Score)	Retrospective	55	271	All Stages	Fuzzy	a, y
							BMI	a, z
							Treatment	a, z
							TNM Stage	b, z
**2012** [24]	Serum CRP and Histological Subtype	5.75 mg/L	Prospective with control	70	53 C:90	All Stages	EC, CRP	-/ y
							ESCC, CRP	-/ x
**Gastro-esophageal**								
**2006** [211]	IL-1β, IL-6, IL-8, TNF-α mRNA, Protein: Tumoral & Systemic Levels	>10	Prospective	70	56 + 22 controls	All stages	CRP	b, x
							IL-1β infiltrate	—
**2006** [203]	GPS in Inoperable Cancer	>10	Prospective	60	258	All stages	Active treatment, GPS	a, z
							Stage TNM	a, z
							Treatment	a, y
							Supportive treatment Stage	a, x
**1982** [212]	Postoperative Survival and Pretreatment CEA, Albumin, CRP, ACT, AGP	>10	Prospective	55	104	All stages	ACT	—
							CRP	—
							AGP	—
**2007** [108]	Factors Predictive of Death. Risk Prediction Model	5mg/L	Prospective	70	220	All Stages	CRP	a, x
							WL Rate	a, x
							Karnofsky	b, y
							Stage IV	c, z
**2008** [127]	GPS & ECOG-PS: Survival & Treatment Response	10mg/L	Prospective	60	65	All Stages	GPS	a, z
**2010** [213]	Pre-treatment Clinical Prognostic Factors and Survival	10mg/L (GPS)	Retrospective	60	217	All Stages	mGPS	b, z
							TNM Stage	a, z
							Position	a, z
							Age	a, z
**2011** [214]	Tumor proliferation, Systemic Inflammatory Response and Survival	10mg/L mGPS	Prospective	60	100	All Stages	mGPS	b, z
							LN ratio	a, x
							Tumor Differentiation	b, z
							Klintrup	b, x
							Ki-67	a, x
**Gastric**								
**1983** [215]	Preoperative CEA, CRP, GGT, PHI, Pseudouridine, ACT, AAG	>20	Prospective	70	200 + 73 C	All stages	Gastric CRP	—
							Colorectal Dukes	—
**2010** [102]	Preoperative CRP and Survival	3mg/L	Prospective with controls	80	170, C: 405	Resectable	CRP	—/ y
**2010** [216]	Hypoalbuminemia, High CRP and Survival	≤10, >10	Retrospective	60	217	All Stages	Continuous CRP	b, z
							Categorical CRP	b, z
							Act. Pall.	b, z
							TNM IV	b, z
**2011** [217]	Preoperative CRP	5mg/L	Retrospective	60	204	Curative	Preoperative CRP	b, x
							Tumor Stage	b, x
							LN Invasion	b, x
**2011** [218]	GPS and Prognosis	10mg/L TGPS, 5mg/L MGPS	Retrospective	65	232	Resectable	TGPS	b, x
							Stage	d, z
							MGPS	b, x
**2011** [219]	Peritoneal Dissemination and Prognosis	20mg/L	Retrospective	55	79	Metastasis	CRP	b, y
							Albumin	a, x
							Ascites	a, x
							ECOG PS	a, z
**2011** [97]	Clinical Status, Laboratory factors and Survival	10mg/L GPS	Retrospective	55	402	Metastasis	GPS 1	a, z
							GPS 2	a, z
							ECOG PS	a, x
**2012** [220]	CRP & Potential Prognostic Factors	10mg/L	Retrospective	50	61	Metastasis	CRP	b, y
							Gender	b, y
**2012** [221]	mGPS and Prognosis	10mg/L mGPS	Retrospective	55	1710	All Stages	mGPS	a, y
							Tumor Stage	b, z
							Age	a, y
**2012** [222]	GPS and Survival	10mg/L GPS	Retrospective	50	83	Advanced	GPS	a, y
							Age	b, y
**2012** [223]	NLR and mGPS in Advanced Stage	10mg/LmGPS	Retrospective	55	104	Unresectable	mGPS1	a, z
							mGPS2	a, y
							NLR	a, x
							LN Mets	a, y
**2012** [228]	GPS before curative surgery and survival	10mg/L GPS	Retrospective	55	366	Specific stages: TNM Stage II & III	GPS	b, z
							LN Metastasis	a, z
							LN Invasion	b, x
							Invasion Depth	b, y
**2012** [128]	Markers of Systemic Inflammatory Response and Prognosis	10mg/L mGPS	Prospective	60	120	All Stages	mGPS	b, z
							LN Ratio	b, z
**Gastric + Colorectal**								
**2000** [224]	Metastasis, KPS Anthropometry, Appetite, Blood Markers, and CRP	>10	Prospective	70	91	Locally Advanced or Metastatic	CRP	—, z
							KPS	—, y
							Mets	—, x
**Other Gastrointestinal**								
**2004** [225]	Albumin, CRP	>10	Retrospective	60	165	Advanced	GPS	—, z
							Tumor type	—, y
							Age	—, x
**Tumor Recurrence**								
**Colorectal**								
**1995** [226]	APR (CRP)	>5	Prospective	70	36	Duke’s B/C	CRP	—
**2001** [227]	CEA, CA19-9 and CRP	>0.5 ng/ml	Prospective	60	82	Dukes’ A, B,C	CRP	—
							CA 19–9	—
**2007** [178]	Pre-operative Inflammatory Response Scoring System & Recurrence	10mg/L	Prospective with Control	75	560	Resectable	IRT	a, z
							Number of metastasis	b, x
**Esophagus**								
**2003** [228]	Outcomes Post-Recurrence	≥10	Prospective + Retrospective	55	258	All stages	S-p53-Abs	d, z
							CRP	c, y
**2011** [208]	GPS and Recurrence in Homogenous Esophageal Cancer	10mg/L GPS	Retrospective	65	495	Resectable	SCC: GPS1	b, y
							GPS2	b, z
							Adeno: GPS1	a, x
							GPS2	b, z
**Gastric**								
**2011** [97]	Laboratory Factors and Progression	10mg/L GPS	Retrospective	55	402	Metastasis	CRP	a, z
							ECOG PS	a, x
							Bone Metastasis	a, y
**2012** [220]	CRP and Gastric Cancer Progression	10mg/L	Retrospective	50	61	Metastasis	CRP	—/ z
**2012** [229]	Inflammation Based Prognostic Score and Recurrence	5mg/L	Retrospective	60	197	Locally Advanced	Inflammatory Score	a, x
							TNM	d, z
							Serous Invasion	a, x
**2012** [222]	GPS and Recurrence	10mg/L GPS	Retrospective	50	83	Advanced	GPS	a, y
**Gastric + Colorectal**								
**2000** [224]	CRP, Metastasis, KPS and Blood Markers	>10	Prospective	70	91	Locally Advanced or Metastatic	CRP	—, z
							KPS	—, y
							Mets	—, x
**Treatment Response and/or Staging**								
**Colorectal**								
**1995** [230]	Pre-treatment APP (4); Response to Immuno- chemotherapy	>10	Prospective	55	24	Metastatic	CRP	—
							Albumin	—
							α1-AT	—
**2006** [175]	Adjuvant 5-FU Post Resection + Survival	>10	Prospective	60	222	Dukes A, B, C	CRP	c, x
**2011** [193]	mGPS and Response in Potentially Curative Resection	5 mg/L (mGPS)	Retrospective	55	219	Specific Stage: Stage II	mGPS	b, y
							Pathology	b, x
**Esophagus**								
**2005** [201]	Pretreatment CRP in CRT + Survival	≥6	Prospective	65	67 + 20 controls	All stages	CRP	—
**2011** [207]	Locally Advanced Disease Under Induction CRT	3mg/L	Retrospective	55	34	Advanced	CRP (Post CRT)	-/y
**Gastro-esophageal**								
**2008** [127]	GPS, ECOG-PS & Clinical Response	10mg/L	Prospective	60	65	All Stages	GPS	—/x

Notes: (86, 89) are survival studies where treatment response was also an outcome.

* All CRP levels reported in results correspond to serum levels unless otherwise specified.

^#^ Since CRP values are reported in different units, for uniformity purposes we converted all values to mg/L unless otherwise specified.

^ϕ^ Strongest predictors by MVA were stratified by relative risk (RR) and statistical significances (p) as follows:

^a^ RR<2 or >0.5

^b^ RR>2 or <0.5

^c^ RR>5 or <0.2

^d^ RR>10 or <0.1

^x^ p<0.05

^y^ p<0.01

^z^ p<0.001

—Values not reported or no MVA

**Abbreviations**: **AAG**: α1 acid glycoprotein; **α1-AT**: α1 Antitryspsin; **ACE-27**: Adult Comorbidity Evaluation-27: **ACT**: α1 Antichymotryspsin; **ALP**: Alkaline Phosphatase; **APP**: Acute Phase Protein(s); **CEA**: Carcinoembryonic Antigen; **CRT**: Chemoradiotherapy; **FU**: Fluorouracil: **GGT**: Gamma Glutamyl Transferase; **GPS**: Glagcow Prognostic Score; **HGF**: Hepatocyte Growth Factor; **HsP**: Heat Shock Protein; **IAP**: Immunosuppressive Acid Protein; **IL**: Interleukin; **LN**: Lymph Node; **MVA**: Multivariate Analysis; **PAI**: Plasminogen Activator Inhibitor-1; **PH**: Partial Hepatectomy; **PHI**: Phosphohexose Isomerase; **PS**: Performance Status; **RR**: Relative Risk; **SCC**: Squamous Cell Carcinoma; **TNF**: Tumor Necrosis Factor; **↑**: Increase; **↓**: Decrease.

2.2. Treatment response or tumor stage: CRP predicted treatment response in six studies [127, 175, 193, 201, 207, 230] (Table 4). It did not predict stage in one study [57] but this was underpowered (Table 4). Treatment responses were evaluated after curative resection followed by adjuvant 5-Flurouracil (5-FU) in localized GI tumors. Responses after neo-adjuvant chemotherapy, chemo-radiotherapy, and IL-2 infusions (with either 5FU or surgery) were observed in advanced tumors. In 4 of the five studies, high CRP level was associated with poorer responses.

2.3. Tumor recurrence: Ten of the 90 GI studies investigated recurrence as a primary outcome. In six of the ten, high CRP independently predicted recurrence. One study did not [59]; it included both retrospective and prospective cohorts. Furthermore, CRP prediction of recurrence was not the main outcome [226–230] (Table 4). High CRP was a strong predictor of recurrence in the rest of the other studies.

#### 3. Other Solid Tumors

24 studies (each) investigated CRP and prognosis in pancreatic and lung cancer. CRP predicted prognosis in 23 of 24 (96%) studies in pancreatic cancer [68–71], 22 of 24 (92%) in lung cancer [25, 72–74], all 10 in hepatocellular carcinoma (HCC) [75–77], all 5 in melanoma [23, 78], 4 of 7 (57%) in breast cancer [79, 80], 12 of 12 (100%) in bladder cancer [81–83], 7 of 9 (78%) in prostate cancer [84–86] and 21 of 24 (88%) others (cervical cancer, ovarian cancer, bone and soft tissue etc.) [87–91]. 14 of 15 (93%) studies of heterogeneous cancers found high CRP to be a predictor of prognosis [92–94] (Table 2, Fig 2).

### CRP and prognosis by univariate analysis

CRP as a prognostic indicator was investigated as the primary outcome in most of these studies. Eighteen percent of all studies (48 of 271) found CRP prognostic only by univariate analysis. The forty eight consisted of 12 in renal cell carcinoma; 10 in colorectal cancer; 6 in gastroesophageal; 7 in pancreas; 2 each in lung and bladder; 2 in heterogeneous groups; 1 in hepatocellular cancer and 6 in others (ovarian, primary bone and soft tissue cancers, oral squamous cell carcinoma, hepatocellular carcinoma and malignant histiocytoma) [231–246] (S3 Appendix). The median sample size was one hundred fifty five (range 38–9608). Thirty included various disease stages, and another 18 advanced, or metastatic/recurrent disease [59, 63, 66, 95–101]. One had an adequate quality score [102], forty three intermediate. 4 were underpowered [39, 59, 95, 103].

### Negative studies

Overall, CRP was not prognostic in 26 of 271 studies (17 prospective, 9 retrospective) (S4 Appendix). These included 9 in digestive tumors; 5 in renal cell carcinoma; 3 in breast; 2 each in lung and prostate; 1 in pancreas; 1 in heterogeneous and 3 in other cancers patients. Median sample size was one hundred thirty eight (range 31–329). 15 of the 26 included various disease stages [57, 58, 65, 80, 104–110]. The others were resectable/unresectable or advanced/locally advanced and/or metastatic disease [38, 111–113]. Although all had intermediate quality scores, three were also underpowered [38, 57, 112]. In most negative studies, CRP as a prognostic indicator was not the primary outcome measure [247–255] (S4 Appendix).

### Additional parameters used for prognosis

CRP was used alone in 6% (15 of 271). Many studies considered more than one parameter for prognostic purpose. Demographic characteristics (age, gender, sex) were included as prognostic parameters in 66% of studies (170/256). Common clinicopathologic parameters included with CRP were: stage (TNM, Dukes, others) 23% (59/256); metastasis (lymph node, liver, others) 17%; performance status (ECOG, KPS, others) 16%; tumor characteristics (histology, site, diameter, size) 16%; WBC 13%. Biochemical parameters used with CRP (specifically in renal cell carcinoma) were: albumin (alone or as hypoalbuminemia), LDH, and interleukins (IL-6, IL-8, IL-2). In digestive tumors common biochemical parameters used were: albumin (alone or hypoalbuminemia), carcinoembryonic antigen (CEA), cancer antigen 19–9 (CA19-9) and interleukins (IL-6, IL-8, IL-2).

## Discussion

### Summary of evidence

Efforts to improve prognostication in cancer had limited success [114]. The number of cancer prognostic biomarkers validated as clinically useful is small, despite extensive research [115, 116]. Many studies have been underpowered. These studies are also difficult to interpret and compare because of heterogeneous study designs. This has prevented meta-analyses of prognostic biologic markers [4, 117]. We encountered this same difficulty during this systematic review.

Although thirty four percent of the studies (92 of 271) used an elevated CRP cut-off point of >10mg/L, the rest varied. The cut-off value was not reported at all in twenty one studies, and simply as present/absent, or positive/negative in others. Reported cut-off values extended over a wide range: 0.5ng/ml, 1ng/ml, >94nmol/L, >2mg/L, >5mg/L, >8mg/L, > 11 or 12 mg/L, > 35 or 50 mg/L. This made meaningful study comparisons difficult. We tried to standardize if not, cut-off values, then at least the units used. All the studies (except one of the high sensitivity CRP) used CRP. Most (>90%) of CRP levels were reported either in milligram per liter or milligram per deciliter (mg/L or mg/dL).

High sensitivity CRP (hs-CRP), tumoral CRP and CRP kinetics have also been utilized for disease progression and prognosis. Increased hs-CRP has been associated with late recurrence in renal cell carcinoma [119] and with increased mortality in breast cancer [79] and in men with lung cancer [118]. Tumoral CRP (increased locally within the tumor) may be superior to serum CRP for prognosis and recurrence [120]. Determined by CRP gene expression, tumoral CRP values are more personalized and rather a target for individualized therapy [121]. CRP kinetics may predict survival [122], recurrence [55] and clinical course [123] in cancer. Human CRP gene is located on the chromosome 1q21-23, spans 1.9 kb and has two exons. CRP gene polymorphism has been associated with increased cancer risk and worse prognosis, mainly in colorectal cancer [124, 125].

Various prognostic scoring systems and instruments have been developed utilizing CRP along with other clinical parameters. Prognostic Inflammatory Nutritional Index, PINI (CRP, Alpha-1 Acid Glycoprotein, albumin and prealbumin) [126]; Glasgow Prognostic Scale or Modified Glasgow Prognostic Scale (CRP, albumin); [50, 127, 128] Fuzzy Logic Based Prognostic Score (CRP and albumin) [129]; Biomarker Based Score (CRP, albumin, Gamma- Glutamyl Transferase (GGT) and HDL) [130]. GPS/mGPS and Fuzzy score only differ by CRP cutoffs. We included studies which utilized the Glasgow Prognostic Score or modified Glasgow Prognostic Score, as identified by the search criteria. We have not included studies which utilized Fuzzy score except for discussion purpose. The dominant biochemical component in both GPS and mGPS is CRP [131, 132]. One study defined mGPS as an Inflammation Based Index (IBI) and utilized it as a validated prognostic index for HCC [76].

CRP is a non-specific marker of inflammation. It can be elevated for many reasons: infection, invasive procedures, or medications [133, 134]. Inadequate screening for known non-cancer CRP-modifying factors may have significantly influenced values. In addition, it is accepted that sensitivity, specificity, positive and negative predictive values should be used to validate and compare any test against a gold standard [135]. Only two studies reported this data.

Inflammatory cells are tumor promoters. They produce an attractive environment for tumor growth, induce DNA damage, promote angiogenesis, and favor neoplastic spread and metastasis [92], and so may affect prognosis [17]. Several explanations exist for the proposed relationship between inflammation and the natural history of cancer. First, tumor growth itself can cause inflammation of surrounding tissue and increase plasma CRP [136]. Second, tumor cells produce various cytokines and chemokines that attract leukocytes. Some cancer cells express CRP and secrete interleukin-6 and interleukin-8, which stimulate liver CRP production [14, 136]. Studies have also shown that IL-6 blocks p-53 induced apoptosis. CRP-positivity develops a favorable microenvironment for the tumor cells through acute inflammatory cytokine network system maintenance [73]. Finally, CRP may be part of the host tumor immune response [136]. Evidence also suggests a causal role for chronic inflammation in several malignancies [14, 136, 137].

Cytokines and their surrogate markers (like CRP and IL-6 receptor) can be elevated both locally and systemically in different solid tumors. In renal cell carcinoma, the imbalance between pro-inflammatory cytokines and their anti-inflammatory counterpart is the therapeutic rationale behind immunotherapy [51, 138]. Colorectal cancer seems linked to chronic inflammation (both local and systemic) from genesis to progression [139]. Similar observations have been made in pancreatic [16] and lung cancers [140]. Those tumors are also highly associated with the cancer anorexia-cachexia syndrome, which itself may in part be due to inflammation [141].

The role of CRP as a prognostic marker for cardiovascular risk is widely known. Although studies have included large sample sizes, some skepticism still exists [12, 133]. It is noteworthy that a recently published study of 270,000 hospital patients, showed that high CRP levels not only predicted all-cause mortality (compared to the low/or normal CRP group), but also higher cancer mortality [142]. This study was retrospective and may have suffered from selection bias; those who had CRP measured were sicker and so had a higher risk of death.

In our review, most studies (over half) which met inclusion and quality criteria were in gastrointestinal and renal cell carcinoma. We were surprised not to see more investigations in lung and pancreatic cancer, since they are often considered clinically to have an inflammatory component. This was perhaps influenced by publication bias and selective reporting, i.e. positive studies published while some negative studies may not even be submitted for publication [143].

In our review, CRP appeared to be a valuable prognostic predictor particularly in digestive tumors and renal cell carcinoma. It may also help predict tumor recurrence and treatment response in those diseases. CRP was compared to other clinical and biochemical factors in these tumors. In renal cell carcinoma, grade, TNM staging, albumin and lactate dehydrogenase (LDH), were among the strongest prognostic predictors by multivariate analysis. Age, Dukes’ stage, albumin, carcinoembryonic antigen (CEA) and the Glasgow Prognostic Score were amongst those in gastrointestinal tumors.

Does CRP add any extra information to these other predictors? CRP can be easily and reliably measured. However, it is a non-specific marker. Levels can rise for numerous reasons independent of the cancer; this also reduces the value of single versus serial CRP measurements. Longitudinal studies of CRP values were largely absent. Since CRP cut-off points differed among studies, and the sensitivity and specificity comparisons with different prognostic variables were unreported, it was impossible to conclude with certainty whether CRP was a better predictor than others. In the negative studies, the role of CRP as a prognostic predictor was not the primary outcome and most were underpowered to detect a difference.

### Limitations

This review had several limitations. Survival and treatment outcomes in the literature were defined and reported inconsistently. Identification of studies depended on CRP being indexed, so we may have been more likely to identify positive studies. Quality assessment was conducted with no cross-validation. The QA system had been piloted on 10 studies picked randomly before the review. This showed it could distinguish between studies in the three QA categories (see Appendices). No meta-analysis or direct study comparisons were done because of the methodological issues described. For similar reasons side by side study comparisons were not possible.

## Conclusions

Increased CRP level predicted prognosis in most (90%) of the studies in solid tumors which met inclusion and quality criteria identified in this systematic review. More than half of all studies (52%) were in gastrointestinal malignancies or renal cell carcinoma. High CRP predicted prognosis in most reports (90%) in these two tumor groups. In addition CRP predicted prognosis in most reports in other solid tumors, so it may also be a clinically useful predictor in lung, pancreas, hepatocellular, and bladder cancers. CRP appeared to be a valuable (and probably under-recognized) prognostic predictor in these tumors. It may also have a role in determining treatment response, and tumor recurrence. The balance of evidence supports wider (and perhaps routine) use of CRP by oncologists for staging, assessment of tumor response and prognostication in at least these two tumor types. These conclusions and recommendations must be tempered by the intermediate quality of most studies.

Despite some methodological issues, CRP appears valuable to help predict prognosis and other important clinical outcomes in many solid tumors. Better quality prospective longitudinal studies on the role of CRP as a prognostic indicator are needed to confirm these observations.

## Supporting Information

S1 Appendix A: Study Quality Assessment Scoring System. (DOCX)

S2 Appendix B: Study Quality Assessment Rating. (DOCX)

S3 Appendix C: CRP as a Prognostic Predictor (Univariate Analysis. (DOCX)

S4 Appendix D: CRP not a Prognostic Indicator. (DOCX)

S1 Data (XLS)

S2 Data (XLS)

S1 PRISMA Checklist (DOC)
