# Supplementary material for: C-Reactive Protein Is an Important Biomarker for Prognosis Tumor Recurrence and Treatment Response in Adult Solid Tumors: A Systematic Review
Source: PLoS One. 2015 Dec 30;10(12):e0143080. doi: 10.1371/journal.pone.0143080 (PMC4705106; doi:10.1371/journal.pone.0143080)
Supplement: S1 Appendix — (DOCX) [file pone.0143080.s001.docx]

**Supporting Information A: Study Quality Assessment Scoring System**

| **Criteria** | **Specifics** | **Score** |
| --- | --- | --- |
| **Study Design**  **(pick one only)** | Prospective Longitudinal with CG | 2.0 |
|  | Prospective Longitudinal without CG | 1.5 |
|  | Prospective, Cross-Sectional design | 1.25 |
|  | Retrospective with CG | 1.0 |
|  | Retrospective without CG | 0.5 |
|  | Case Series | 0.0 |
| **Patient Selection** | - Sample well defined  - Sample not well defined | 0.5  0.0 |
|  | - Similar point in the course of the disease  - Different disease stages | 0.5  0.0-0.5 |
|  | **Power analysis described:**   - Yes - No | 0.5  0.0 |
|  | **Sample size**:   - Well powered - Not well powered | 0.5  0.0 |
|  | Total Score |  |
| **Prognostic variables** | **Outcome measure** (pick one):  - Well defined  - Not well defined | 1.0  0.0 |
|  | **Measurements** (circle one):   - Objective - A combination of both - Subjective | 1.0  0.5  0.0 |
|  | Total Score |  |
| **Follow up**  (N/A if Cross-sectional/ retrospective  design) | **Duration of follow up** (pick one):   - Adequate - Not adequate or not stated | 1.0  0.0 |
|  | **Drop–outs** (pick one):  - None   - Reported and explained - Not reported or not explained | 1.0  0.5  0.0 |
|  | Total Score |  |
| **Analysis** | **Confounding factors** (pick one):  - None   - Comparable across groups or adjusted for - Comparable for some or adjusted for some factors but not all - Not comparable or not reported or not adjusted for | 2.0  1.0  0.5  0.0 |
| **Total** | Sum of all criteria scores |  |
|  | % of maximum score |  |
