# Supplementary material for: C-Reactive Protein Is an Important Biomarker for Prognosis Tumor Recurrence and Treatment Response in Adult Solid Tumors: A Systematic Review
Source: PLoS One. 2015 Dec 30;10(12):e0143080. doi: 10.1371/journal.pone.0143080 (PMC4705106; doi:10.1371/journal.pone.0143080)
Supplement: S2 Appendix — (DOCX) [file pone.0143080.s002.docx]

**Supporting Information B: Study Quality Assessment Rating**

| **% of maximum score** | **Rating** |
| --- | --- |
| **80-100%** | **Adequate** |
| **≥50 but <80%** | **Intermediate** |
| **< 50%** | **Inadequate** |
