# Supplementary material for: C-Reactive Protein Is an Important Biomarker for Prognosis Tumor Recurrence and Treatment Response in Adult Solid Tumors: A Systematic Review
Source: PLoS One. 2015 Dec 30;10(12):e0143080. doi: 10.1371/journal.pone.0143080 (PMC4705106; doi:10.1371/journal.pone.0143080)
Supplement: S3 Appendix — (DOCX) [file pone.0143080.s003.docx]

**Supporting Information C: CRP as a Prognostic Predictor (Univariate Analysis)**

| **Cancer**  **Type** | **Publication Year**  **(Reference)** | **Main Outcome** | **CRP cut-offs***  **(mg/L)^#^** | **Study Design** | **Quality**  **Score** | **Sample Size** | **Disease Stage** | **Best Predictors^+^**  **By Univariate** |
| --- | --- | --- | --- | --- | --- | --- | --- | --- |
| **Renal** | **1995**  **[**[**29**](#_ENREF_29)**]** | Common inflammatory parameters on prognosis | >10 | Prospective | 65 | 170 | All | Stage d, z  Grade b, x  ESR a, x |
|  | **1997**  **[**[**30**](#_ENREF_30)**]** | Predictive clinical and histological variables | Present or Absent | Retrospective | 55 | 320 | All | M stage d, z  N Stage b, x  Age a, x |
|  | **2000**  **[**[**31**](#_ENREF_31)**]** | Prognostic determinants | Negative or Positive | Retrospective | 55 | 62 | All | M classification d,z  N Classification d,x  ALP d,y |
|  | **2001**  **[**[**32**](#_ENREF_32)**]** | Predictive value of ferritin, ESR, CRP, BPF, IAP | >5 | Retrospective | 65 | 92 | All | Ferritin c, y |
|  | **2008**  **[**[**34**](#_ENREF_34)**]** | IVC Thrombus & Prognosis | 6 mg/L | Retrospective | 55 | 46 | All | CRP b, x  Fever b, x  Grade b, x  LN Mets c, z |
|  | **2009**  **[**[**33**](#_ENREF_33)**]** | Prognosis in worst nuclear grade component | 10 mg/L | Retrospective | 50 | 314 | All | CRP --, y  Tumor size --,y  MVI + --, z  Mets + --, z  Nodes + --, x |
|  | **2009**  **[**[**233**](#_ENREF_233)**]** | Prognosis: Molecular markers + other markers | Normal v. abnormal | Prospective | 50 | 40 | All | CRP b, x  KPS b, x  Mets +/- c, y  Ki-67 b, x  Bcl-2 b, x |
|  | **2010**  **[**[**35**](#_ENREF_35)**]** | Serum Amyloid A, peptide fragments & prognosis | Contin. | Retrospective | 60 | 119 | All | CRP b, z  SAA b, z  M stage d, z  Diameter a, z  MVI d, z  Necrosis c, z |
|  | **2010**  **[**[**234**](#_ENREF_234)**]** | NLR as a predictor of survival | 3 mg/L | Retrospective | 55 | 192 | All | CRP --/ x  NLR --/ x  Lymphocytes --/ x  T stage --/ z |
|  | **2011**  **[**[**36**](#_ENREF_36)**]** | CRP & IL-6 on Anemia & Survival | 7 mg/L | Retrospective | 50 | 86 | All | CRP b, z  EPO b, z  Neopterin b, y  Anemia b, z |
|  | **2011**  **[**[**37**](#_ENREF_37)**]** | Prognosis | Contin. | Prospective | 55 | 95 | All | CRP --, z  Tumor size --, y  CRP stain --, x  Furhman --, y  T stage --, z |
|  | **2012**  **[**[**160**](#_ENREF_160)**]** | Prognosis in curative nephrectomy | 10 mg/L | Prospective | 70 | 169 | All | CRP a, z  Necrosis b, z  Fuhrman a, z  Tumor size a, z  T stage a, z |
| **Gastric/ Gastro-Oesophageal** | **2010**  **[**[**215**](#_ENREF_215)**]** | Pre-treatment clinical factors and survival | 5 mg/L | Prospective | 60 | 217 | All | CRP --, z  Age --, y  TNM --, z  WL --, y  mGPS --, z  Treatment --, z |
|  | **2010**  **[**[**103**](#_ENREF_103)**]** | Clinical-pathologic association and prognosis | 3 mg/L | Prospective | 80 | 170  + 405  (Healthy Control) | Resectable | CRP --, z  Lym. Inv --, y  p Stage --, x  Mets + --, x  Tum depth --, z  Wall Inv. --, z |
|  | **2011**  **[**[**98**](#_ENREF_98)**]** | Clinical status, Lab factors & Overall survival | 10 mg/L | Retrospective | 55 | 402 | Metastasis | CRP --, z  Mets+ --, x  Albumin --, z  ECOG --, y |
|  | **2012**  **[**[**225**](#_ENREF_225)**]** | Inflammation based prognostic score & advanced cancer | 10 mg/L | Retrospective | 55 | 104 | Advanced | CRP a, z  NLR a, y  ALP a, y  Albumin b, y  Bone Mets b, y  LN Mets a, y |
|  | **2012**  **[**[**132**](#_ENREF_132)**]** | Prognostic Score and Outcome Prediction | 5 mg/L | Retrospective | 55 | 271 | All | CRP --, z  TNM --, z  Treatment --, z  Albumin --, z  Bilirubin --, z  Platelet --, x |
|  | **2012**  **[**[**25**](#_ENREF_25)**]** | Prognosis in oesophageal cancer subtypes | 5.75 mg/L | Prospective with control | 65 | 53, 90 (C) | All | CRP --, y  Mets + --, z  TNM --, z |
| **Colorectal** | **1994**  **[**[**60**](#_ENREF_60)**]** | Blood factors (38 variables) in prognosis | >10 | Prospective | 55 | 100 | Recurrence or Metastasis | LDH b, y  Liver mets b, x  α-1 globulin a, y  CEA a, x |
|  | **2001**  **[**[**27**](#_ENREF_27)**]** | Pre-/post-operative APP and disease-specific mortality | >10 | Prospective | 75 | 202 | All | Duke’s stage --, z  CEA>60U/L --, y |
|  | **2003**  **[**[**171**](#_ENREF_171)**]** | Clinico-pathological and preoperative CRP | >10 | Prospective | 65 | 150 | All | Stage b, z |
|  | **2003**  **[**[**68**](#_ENREF_68)**]** | Preoperative CRP in disease specific mortality | >8 | Prospective | 60 | 172 | All | Duke’s stage b, z |
|  | **2007**  **[**[**63**](#_ENREF_63)**]** | Tumor diameter, CRP & survival | 10 mg/L | Retrospective | 60 | 227 | All | CRP --,x  Age --, x  TNM --, y |
|  | **2010**  **[**[**235**](#_ENREF_235)**]** | Serum Retinol, Alpha-Tocopherol & Systemic Inflammation on Survival | 24 mg/L | Prospective | 55 | 25 | Metastasis | CRP --, x  ↓ Retinol --, x |
|  | **2011**  **[**[**64**](#_ENREF_64)**]** | Peritoneal Carcinomatosis: Survival | 35 mg/L | Retrospective | 50 | 50 | Advanced | CRP --, z |
|  | **2012**  **[**[**65**](#_ENREF_65)**]** | Tumor markers and Survival | 5 mg/L | Prospective | 55 | 106 | All | CRP a, z  CEA a, z  CA 19-9 a, z  TPA a, z  ALP a, y  WBC a, y  Age a, x |
|  | **2012**  **[**[**67**](#_ENREF_67)**]** | Clinical Factors & Survival | Contin. | Retrospective | 55 | 169 | Advanced | CRP a, y  NLR a, y  Neutrophil a, y  Lymphocyte a, y  Tumor path a, z |
|  | **2012**  **[**[**236**](#_ENREF_236)**]** | Inflammation based system & post-operative survival | Contin. | Retrospective | 60 | 271 | All | CRP a, x  Lymphatic+ b, x  Platelet a, x  CEA a, x  TNM b, y |
| **Lung Cancer** | **2008**  **[**[**237**](#_ENREF_237)**]** | CRP and Survival | Contin. | Retrospective | 50 | 292 | Advanced | CRP --, z |
|  | **2011**  **[**[**97**](#_ENREF_97)**]** | Prognostic value of Inflammatory markers | Contin. | Prospective | 60 | 115 | Metastasis | CRP a, x  Albumin a, z  Mets sites b, z  PS b, z  Leptin a, y |
| **Bladder Cancer** | **2012**  **[**[**100**](#_ENREF_100)**]** | Overall survival | 10mg/L | Retrospective | 50 | 67 | Advanced | CRP --, z  GPS --, z  PS --, z  Albumin --, z  Lung Mets --, y  Bone Mets --, y |
|  | **2012**  **[**[**238**](#_ENREF_238)**]** | Pre-operative Prognostic Factors | 5 mg/L | Retrospective | 50 | 189 | Resectable | CRP --, x  Tumor Size --. y  T stage --, z  Hydronep.+ --, z  Hemoglobin --, z  Neutrophil --, z |
| **Pancreatic** | **2001**  **[**[**96**](#_ENREF_96)**]** | Prognostic factors | Not reported | Retrospective | 50 | 193 | Advanced | Chemotherapy a, y  Stage b, x  Appetite a, y |
|  | **2008**  **[**[**239**](#_ENREF_239)**]** | Markers that predict survival | 50 mg/L | Prospective | 55 | 136 | Advanced | CA 19-9 b, x  CEA a, x  Albumin a, y  Leukocytes a, x  Mets+ a, x |
|  | **2011**  **[**[**240**](#_ENREF_240)**]** | Gonadal status, systemic inflammation in nutritional status and prognosis | 10 mg/L | Prospective | 70 | 167 | Advanced | CRP --, y  SHBG --, y  IL-6 --, y |
|  | **2012**  **[**[**241**](#_ENREF_241)**]** | Unresectable Pancreatic Cancer under Treatment: Prognosis | 10 mg/L | Retrospective | 50 | 41 | All | CRP --, x  CA 19-9 --, x  CEA --, x  Lymphocytes --, y  Ascites + --, x  Jaundice+ --, x |
|  | **2012**  **[**[**242**](#_ENREF_242)**]** | Identify survival predictors | 5 mg/L | Retrospective | 50 | 231 | Locally Advanced | CRP --, y  CA19-9 --, x  LDH --, x  Albumin --, x  Bilirubin --, y  WBC --, y |
|  | **2012**  **[**[**243**](#_ENREF_243)**]** | Validation of prognostic indices | <10  10-30  ≥30 mg/L | Retrospective | 55 | 116 | Advanced | CRP --, x  LDH --, y  Bilirubin --, y  ECOG --, z |
|  | **2012**  **[**[**244**](#_ENREF_244)**]** | Inflammation based prognostic scale vs. WBCs scale | 10 mg/L | Retrospective | 50 | 177 | All | CRP a, y  TNM b, z  PI a, z  NLR b, z  Neutrophils b, z  WBC b, z |
| **HCC** | **2012**  **[**[**99**](#_ENREF_99)**]** | TACE therapy, biomarkers and Survival | Contin. | Prospective | 55 | 38 | Advanced | CRP --, x  AFP --, y  Bilirubin --, y |
| **Heterogen.** | **2012**  **[**[**102**](#_ENREF_102)**]** | Systemic Inflammation & Survival | 10 mg/L | Retrospective | 50 | 68 | Advanced | CRP --, z  Albumin --, x  IL-6 --, y  NLR --, x  Site --, x |
|  | **2011**  **[**[**135**](#_ENREF_135)**]** | Inflammation, biochemical parameters, tumor site and survival | 10 mg/L | Retrospective | 60 | 9608 | All | CRP --, z  Albumin --, z  Calcium --, z  Bilirubin --, z  ALP --, z  AST --, z  ALT --, z |
| **Others** | **1996**  **[**[**92**](#_ENREF_92)**]** | Clinical relevance of pre-treatment doubling time of serum markers | Not reported | Prospective | 65 | 132 | All | ALP b, x  (Doubling time) |
|  | **1999**  **[**[**91**](#_ENREF_91)**]** | Preoperative CRP in prognosis | ≥50 | Retrospective | 65 | 120 | All | N/A |
|  | **2002**  **[**[**104**](#_ENREF_104)**]** | CRP as a marker of paraneoplastic syndrome detection and prognosis | >10 | Retrospective | 55 | 46 | All | Stage -- |
|  | **2010**  **[**[**245**](#_ENREF_245)**]** | mGPS & vulvar cancer survival | 10 mg/L | Retrospective | 60 | 93 | All | mGPS --, z  Age --, y  Stage --, z  LN+ --, z |
|  | **2012**  **[**[**90**](#_ENREF_90)**]** | Pre-treatment Level and Overall Survival | 3 mg/L | Retrospective | 55 | 102 | All | Grade (Hist.) --, z  CRP --, y |
|  | **2012**  **[**[**246**](#_ENREF_246)**]** | Investigate Relevant Prognostic Factors | 11 mg/L | Retrospective | 60 | 187 | All | CRP --, z  Hemoglobin --, z  WBC --, z  Grade (Hist.) --, z |

* All CRP levels reported in results correspond to serum levels unless otherwise specified

^#^ Since CRP values are reported in different units, for uniformity purposes we converted all values to mg/L unless otherwise specified

^φ^ Strongest predictors by MVA were stratified by relative risk (RR) and statistical significances (p) as follows:

(a) When 0.5<RR<2

(b) When 2<RR<0.5

(c) When 5<RR<0.2

(d) When 10<RR<0.1

(x) When p<0.05

(y) When p<0.01

(z) When p<0.001

-- Values not reported or no MVA

**Abbreviations**: **ALP**: Alkaline Phosphatase; **APP**: Acute Phase Proteins; **BFP**: Basis Fetoprotein; **CEA**: Carcinoembryonic antigen; **EPO**: Erythropoietin; **ESR**: Erythrocyte sedimentation rate; **HCC**: Hepatocellular Carcinoma; **IAP**: Immunosuppressive acidic protein; **LDH**: Lactate Dehydrogenase; **MVI**: Microvascular Invasion; **N/A**: Not applicable; **NLR**: Neutrophils-Lymphocyte Ratio; **PLR**: Platelet0-Lymphocyte Ratio; **SAA**: Serum Amyloid A; SHBG: Sex Hormone Binding Globulin; **TACE**: Transarterial Chemoembolization;
