# Supplementary material for: C-Reactive Protein Is an Important Biomarker for Prognosis Tumor Recurrence and Treatment Response in Adult Solid Tumors: A Systematic Review
Source: PLoS One. 2015 Dec 30;10(12):e0143080. doi: 10.1371/journal.pone.0143080 (PMC4705106; doi:10.1371/journal.pone.0143080)
Supplement: S4 Appendix — (DOCX) [file pone.0143080.s004.docx]

**Supporting Information D: CRP not a Prognostic Indicator**

| **Cancer Type** | **Pub. Year**  **[Ref.]** | **QA** | **Study Design** | **Main Outcome** | **Sample Size** | **Extent of Disease** | **CRP**  **cut-offs** | **Best Predictor(s)** |
| --- | --- | --- | --- | --- | --- | --- | --- | --- |
| **Renal** | **2002**  **[**[**39**](#_ENREF_39)**]** | SA  60% | PL no CG | Toxicity and efficacy of IL-2, Interferon- and medroxyprogesterone | 42 | Metastatic or locally advanced |  | PS |
|  | **2008**  **[**[**247**](#_ENREF_247)**]** | SA  50% | Rt. no CG | Comprehensive prognostic stratification | 197 | Metastatic | NR | ECOG  N stage  Sarcomatoid Dif. Liver Mets.  Mets. number |
|  | **2011**  **[**[**248**](#_ENREF_248)**]** | SA  60% | Rt. no CG | Prognosis and BMI | 170 | Localized | 3 mg/L | BMI  ECOG PS |
|  | **2011**  **[**[**249**](#_ENREF_249)**]** | SA  55% | Rt. no CG | Prognosis & CD45RO + T cells | 105 | All | Positive v. Negative | Dist. Metastasis  CD45RO+ status |
|  | **2012**  **[**[**121**](#_ENREF_121)**]** | SA  60% | Rt. no CG | Recurrence post-surgery and prognosis | 747 | All | Continu. | BMI  Fuhrman’s Grade  LVI |
| **Digestive** | **1996**  **[**[**58**](#_ENREF_58)**]** | SA  55% | PL + CG | APP and tumor markers (CEA, CA19-9) in preoperative staging | 22 + 9 CT | All stages (Preoperative) | >5mg/L | AGP  CA19-9 |
|  | **2009**  **[**[**250**](#_ENREF_250)**]** | SA  60% | PL + CG | Serum IL-6 & IL-10: Host immunity & Survival | 90 + 6 | All stages | 2 mg/L | Tumor stages  IL-10 |
|  | **2009**  **[**[**251**](#_ENREF_251)**]** | SA  50% | Rt. no CG | Therapeutic outcomes & Prognosis: On CRT | 71 | Unresectable | 10 mg/L | Treatment Res.  PS |
|  | **2009**  **[**[**110**](#_ENREF_110)**]** | SA  75% | PL no CG | IL-6, CRP and disease status & survival | 115 | All stages | 1.45 mg/L | IL-6 |
|  | **2010**  **[**[**59**](#_ENREF_59)**]** | SA  65% | PL+ CG | Pre-operative VEGF, IL-6 & CRP : Prognosis | 132 + 50 | All stages | 5 mg/L | VEGF  CEA  LN status  Age |
|  | **2011**  **[**[**111**](#_ENREF_111)**]** | SA  70% | PL + CG | CEA, CA19-9, IL-6, CRP and Prognosis | 92 + 70 | All stages | 5.2 mg/L | Tumor Stage  CEA  CA19-9 |
|  | **2011**  **[**[**66**](#_ENREF_66)**]** | SA  55% | PL no CG | Systemic Inflammation predicts survival | 152 | All stages | 10 mg/L | Clinical response  LN metastasis |
|  | **2012**  **[**[**252**](#_ENREF_252)**]** | SA  50% | PL no CG | Circulating Cytokines + Prognosis | 77 | Stage III | 20 mg/L | IL-6  Nodal status  LVI |
|  | **2012**  **[**[**253**](#_ENREF_253)**]** | SA  55% | PL no CG | Nuclear Factor-kB (NF-kB) & Prognosis | 115 | All stages | Contin. | NF-kB  LN Ratio |
| **Pancreas** | **2011**  **[**[**254**](#_ENREF_254)**]** | SA  50% | Rt. no CG | Predictive role: CRP, NLR and PLR | 74 | Resectable | Contin. | NLR |
| **Breast** | **1982**  **[**[**105**](#_ENREF_105)**]** | SA  65% | PL no CG | CRP, Lymphocytic infiltrate & Recurrence | 297 | All stages | 10mg/L | None |
|  | **1998**  **[**[**112**](#_ENREF_112)**]** | SA  60% | PL no CG | Pre-tx APP levels in response & survival post neoadjuvant tx | 77 | Locally advanced | >2mg/L | Tumor stage  Node status  Albumin |
|  | **2011**  **[**[**81**](#_ENREF_81)**]** | SA  55% | PL no CG | IL-6 & CRP: Staging & Prognosis | 59 | All stages | 155 mg/L | IL-6 |
| **Prostate** | **1990**  **[**[**106**](#_ENREF_106)**]** | SA  55% | Rt no CG | Disease progression by serum markers | 90 | All stages (operable) | 20mg/L | Neopterin  Tumor grade  Thymidine kinase  PSA |
|  | **1991**  **[**[**107**](#_ENREF_107)**]** | SA  60% | PL no CG | Tumor grade, stage, and short term survival by serum markers | 102 | All stages (at diagnosis) | 20mg/L | Neopterin  Tumor grade  Thymidine kinase  PSA |
| **Lung** | **1985**  **[**[**108**](#_ENREF_108)**]** | SA  70% | PL+ CG | Role in preoperative selection, tx, and prognosis of different serum proteins | 290+35 CT | All stages (Preoperative) | ≥10mg/L | - C3 in operable group  - AGP & RBP in inoperable group |
|  | **2005**  **[**[**113**](#_ENREF_113)**]** | SA  55% | PL + CG | Role of IL-6, TNF-α, CRP, and leptin in diagnosis and prognosis | 28+ 15 CT | Advanced | NR | Stage of disease  LDH  PS  Responsiveness to chemotherapy |
| **Heterog.** | **2010**  **[**[**255**](#_ENREF_255)**]** | SA  50% | PL no CG | Prognostic factors in terminally ill | 209 | NR | 103 mg/L | ECOG  WBC  High Bilirubin  High Creatinine  High LDH |
| **Others** | **2008**  **[**[**256**](#_ENREF_256)**]** | SA  50% | Rt. no CG | Prognosis | 67 | All stages | 5 mg/L | Tumor stage  LN involvement  Age |
|  | **2010**  **[**[**257**](#_ENREF_257)**]** | SA  60% | Rt. no CG | Prognosis | 278 | NR | 5 mg/L | None |
|  | **2011**  **[**[**258**](#_ENREF_258)**]** | SA  50% | PL + CG | Markers of Inflammation, Angiogenesis or Coagulation on Outcome | 47 + 60 | Resectable | 2.4 mg/L | None |

**PL no CG**: Prospective Longitudinal, no Control Group

**PL + CG**: Prospective Longitudinal, with Control Group

**AGP**: α_1_-acid glycoprotein; **APP**: Acute phase protein; **CD**: Cluster of Differentiation; **Continu.**: Continuous (variable); **CRT**: Chemoradiotherapy; **CT**: Control; **IL**: Interleukin; **LDH**: Lactate Dehydrogenase; **LN**: Lymph Node; **LVI**: Lympho vascular invasion; **NLR**: Neutrophil to Lymphocyte Ratio; **NR**: Not Reported; **PLR**: Platelet to Lymphocyte Ratio; **PS**: Performance Status; **PSA**: Prostate Specific Antigen; **QA**: Quality Assessment; **Rt**: Retrospective; **RBG**: Retinol-binding protein; **RCC**: Renal Cell Carcinoma; **SA**: Somewhat Adequate; **Tx**: Treatment or Therapy; **VEGF**: Vascular Endothelial Growth Factor;
