# Supplementary material for: C-Reactive Protein Is an Important Biomarker for Prognosis Tumor Recurrence and Treatment Response in Adult Solid Tumors: A Systematic Review
Source: PLoS One. 2015 Dec 30;10(12):e0143080. doi: 10.1371/journal.pone.0143080 (PMC4705106; doi:10.1371/journal.pone.0143080)
Supplement: S1 PRISMA Checklist — (DOC) [file pone.0143080.s007.doc]

| **Section/topic** | **#** | **Checklist item** | **Reported on page #** |
| --- | --- | --- | --- |
| **TITLE** | | |  |
| Title | 1 | C-REACTIVE PROTEIN IS AN IMPORTANT BIOMARKER FOR PROGNOSIS, TUMOR RECURRENCE AND TREATMENT REPONSE IN ADULT SOLID TUMORS: A SYSTEMATIC REVIEW |  |
| **ABSTRACT** | | |  |
| Structured summary | 2 | A systematic literature review was done to determine the relationship between elevated CRP and prognosis in people with solid tumors. C-reactive protein (CRP) is a serum acute phase reactant and a well-established inflammatory marker. We also examined the role of CRP to predict treatment response and tumor recurrence. MeSH (Medical Subject Heading) terms were used to search multiple electronic databases (PubMed, EMBASE, Web of Science, SCOPUS, EBM-Cochrane). Two independent reviewers selected research papers. We also included a quality Assessment (QA) score. Reports with QA scores <50% were excluded. PRISMA (Preferred Reporting Items for Systematic Reviews and Meta-Analysis) methodology was utilized for this review. 271 articles were identified for final review. There were 45% prospective studies and 52% retrospective. 264 had intermediate QA score (≥50% but <80%); Seven were adequate (80% -100%); A high CRP was predictive of prognosis in 90% (245/271) of studies - 80% of the 245 studies by multivariate analysis, 20% by univariate analysis. Many (52%) of the articles were about gastrointestinal malignancies (GI) or kidney malignancies. A high CRP was prognostic in 90% (127 of 141) of the reports in those groups of tumors. CRP was also prognostic in most reports in other solid tumors primary sites. A high CRP was associated with higher mortality in 90% of reports in people with solid tumors primary sites. This was particularly notable in GI malignancies and kidney malignancies. In other solid tumors (lung, pancreas, hepatocellular cancer, and bladder) an elevated CRP also predicted prognosis. In addition there is also evidence to support the use of CRP to help decide treatment response and identify tumor recurrence. Better designed large scale studies should be conducted to examine these issues more comprehensively. |  |
| **INTRODUCTION** | | |  |
| Rationale | 3 | CRP is an acute phase reactant, which reflects tissue injury. In chronic inflammatory diseases, serial CRP levels have been correlated with disease severity, and response to therapy. CRP is a stable downstream marker of inflammation, unlike the pro-inflammatory cytokines, which have short (minutes) half-lives. Chronic inflammation has been linked to cancer at tumor initiation, but may also be involved with invasive potential and disease progression. A relationship has been proposed between systemic inflammation, cancer symptoms and prognosis. Our systematic review looked at role of CRP in solid tumor prognostication. |  |
| Objectives | 4 | We describe the results of a systematic review of the relationship between elevated serum CRP and life expectancy in people with solid tumors. We also examined its role to help predict treatment response and risk of tumor recurrence. |  |
| **METHODS** | | |  |
| Protocol and registration | 5 | No review protocol exists for this systematic review. |  |
| Eligibility criteria | 6 | We limited articles to those in English. Original reports of any studies of solid malignancies in adults were scrutinized. All study designs were included. The following articles were excluded: all non-English literature, basic research, all pediatric and hematological malignancies, and studies where prognostic parameters were not assessed, or serum CRP levels not measured. Editorial letters and comments were also excluded. Review papers were consulted, but for discussion purposes only. |  |
| Information sources | 7 | Electronic databases included: PubMed (1966 to December 2012); EMBASE (1988 to 2012); Web of Science (1980 to 2012); SCOPUS (1965 to 2012); and the EBM-Cochrane Central Register of Controlled trials and EBM-Cochrane Database of Systematic Reviews (Up to 2012). The search was repeated at the end of data analysis. |  |
| Search | 8 | The following search strategy was applied to Pubmed databse:  ((crp[All Fields] OR ("c-reactive protein"[MeSH Terms] OR ("c-reactive"[All Fields] AND "protein"[All Fields]) OR "c-reactive protein"[All Fields] OR "c reactive protein"[All Fields])) AND ("neoplasms"[MeSH Terms] OR "neoplasms"[All Fields] OR "cancer"[All Fields])) AND (((("prognosis"[MeSH Terms] OR "prognosis"[All Fields]) OR ("mortality"[Subheading] OR "mortality"[All Fields] OR "survival"[All Fields] OR "survival"[MeSH Terms])) OR ("survival rate"[MeSH Terms] OR ("survival"[All Fields] AND "rate"[All Fields]) OR "survival rate"[All Fields])) OR ("treatment outcome"[MeSH Terms] OR ("treatment"[All Fields] AND "outcome"[All Fields]) OR "treatment outcome"[All Fields])) AND (("1966/01/01"[PDAT] : "2013/12/31"[PDAT]) AND English[lang]) AND "humans"[MeSH Terms] |  |
| Study selection | 9 | We met with a qualified medical librarian at our institution before and after the literature search to discuss and adjust our search strategy. The first literature screen was based on article title. If that was irrelevant, the abstract was also reviewed before an exclusion decision. Abstracts (and when necessary the full text) of the remaining articles were then assessed. |  |
| Data collection process | 10 | Data were extracted using a custom designed extraction sheet (till 2010) and later utilizing the Research Electronic Data Capture (REDCap) forms with same sheet details. REDCap is a secure web application to create and manage data. |  |
| Data items | 11 | Author name, publication date, study title, study design, quality assessment score and grade, number of patients and controls (when available), cancer type, extent of disease, main outcome, main results related to prognosis or treatment outcomes, CRP cut-off points, assay method, mean CRP value, survival definition, median survival duration, CRP sensitivity and specificity as a predictor of prognosis, treatment outcomes or recurrence, other parameters assessed for prognosis, strongest predictors of prognosis, statistical analysis used, and possible co-morbid contributors to increased CRP levels other than cancer (like infection, chemo- or radiotherapy, surgery). |  |
| Risk of bias in individual studies | 12 | The reviewer team met periodically to discuss reasons of exclusion or inclusion of selected papers. Retained articles were then subjected to quality assessment (QA). Studies with QA scores >50% were included in the review. |  |
| Summary measures | 13 | For each study article we estimated minimum sample sizes necessary to detect a difference at P ≤ 0.05. We used the general rule of an n = 10 per variable. The estimated minimum sample size was compared to the actual size of the study. Studies with insufficient sample sizes were considered underpowered. Predictors by multivariate analysis (MVA) were stratified by relative risk (RR) and statistical significance (p-value). |  |
| Synthesis of results | 14 | A quality assessment (QA) system based on existing guidelines for observational cohort prognostic studies was developed. The tool combined five criteria: Study design, Patient selection, Prognostic variables, Follow-up, Data analysis: A score of 0-2 was assigned to each - if a study met the conditions in full (score of 2), partially (score of 1), or not at all (score of 0). The total was expressed as a percentage of the maximum possible score. A score of 80-100% was ranked as an adequate study; ≥50 but <80% as intermediate; articles that scored <50% were considered inadequate and excluded from the review. |  |

Page 1 of 2

| **Section/topic** | **#** | **Checklist item** | **Reported on page #** |
| --- | --- | --- | --- |
| Risk of bias across studies | 15 | The studies with borderline QA score and with risk of bias were evaluated by the reviewers and only included if all approved. |  |
| Additional analyses | 16 | No additional analysis was done. |  |
| **RESULTS** | | |  |
| Study selection | 17 | The search identified three thousand and eighty nine citations: 1466 in PubMed, 802 in Web of Science, 320 in SCOPUS, 311 in EMBASE and 190 in the Cochrane database. After removal of duplicates, 1526 remained. Irrelevant studies were then removed. These included those where survival or prognosis was not an outcome, studies where CRP was not studied as a prognostic marker, animal/cell-line based studies, letters and editorials, and those that did not fit our inclusion criteria. Seven hundred thirty one papers were left. Next, three hundred studies in hematologic malignancies, 10 non-English articles and 42 pediatric reports were removed. Subsequent to the quality assessment (QA) of the 379 studies retained, thirty five (ten prospective, twenty five retrospective) were inadequate by QA score and excluded. Then 271 research studies and 73 review papers remained (Figure 1). |  |
| Study characteristics | 18 | Author name, publication date, study title, study design, quality assessment score and grade, number of patients and controls (when available), cancer type, extent of disease, main outcome, main results related to prognosis or treatment outcomes, CRP cut-off points, assay method, mean CRP value, survival definition, median survival duration, CRP sensitivity and specificity as a predictor of prognosis, treatment outcomes or recurrence, other parameters assessed for prognosis, strongest predictors of prognosis, statistical analysis used, and possible co-morbid contributors to increased CRP levels other than cancer (like infection, chemo- or radiotherapy, surgery). |  |
| Risk of bias within studies | 19 | The reviewer team periodically met to discuss reasons of exclusion or inclusion of selected papers. Retained articles were then subjected to quality assessment (QA). Studies with QA scores >50% were included in the review. |  |
| Results of individual studies | 20 | This information has been summarized in table 2 and table 3 of the manuscript. |  |
| Synthesis of results | 21 | The cancer primary sites and study designs of retained articles varied. A meta-analysis was therefore inappropriate. |  |
| Risk of bias across studies | 22 | The reviewers meet periodically to discuss the risk of bias across studies. The decision to include the study was based on consensus. |  |
| Additional analysis | 23 | No additional analysis was done. |  |
| **DISCUSSION** | | |  |
| Summary of evidence | 24 | Increased CRP level predicted prognosis in most of the studies in solid tumors which met inclusion and quality criteria identified in this systematic review. More than half of all studies were in gastrointestinal malignancies or renal cell carcinoma. High CRP predicted prognosis in most reports in these two tumor groups. CRP appeared to be a valuable (and probably under recognized) prognostic predictor in these tumors. |  |
| Limitations | 25 | This review had several limitations. Survival and treatment outcomes in the literature were defined and reported inconsistently. Identification of studies depended on CRP being indexed, so we may have been more likely to identify positive studies. Quality assessment was conducted with no cross-validation. |  |
| Conclusions | 26 | Increased CRP level predicted prognosis in most (90%) of the studies in solid tumors which met inclusion and quality criteria identified in this systematic review. More than half of all studies (52%) were in gastrointestinal malignancies or renal cell carcinoma. High CRP predicted prognosis in most reports (90%) in these two tumor groups. CRP appeared to be a valuable (and probably under recognized) prognostic predictor in these tumors. |  |
| **FUNDING** | | |  |
| Funding | 27 | No external funding was utilized to conduct this systematic review. |  |

*From:*  Moher D, Liberati A, Tetzlaff J, Altman DG, The PRISMA Group (2009). Preferred Reporting Items for Systematic Reviews and Meta-Analyses: The PRISMA Statement. PLoS Med 6(6): e1000097. doi:10.1371/journal.pmed1000097

Page 2 of 2
